# The Axonal Motor Neuropathy-Related HINT1 Protein Is a Zinc- and Calmodulin-Regulated Cysteine SUMO Protease

**DOI:** 10.1089/ars.2019.7724

**Published:** 2019-07-17

**Authors:** Elsa Cortés-Montero, María Rodríguez-Muñoz, Pilar Sánchez-Blázquez, Javier Garzón

**Affiliations:** Neuropharmacology, Instituto Cajal, Consejo Superior de Investigaciones Científicas (CSIC), Madrid, Spain.

**Keywords:** HINT1, desumoylase, zinc, nitric oxide, cysteine oxidation, calmodulin

## Abstract

***Aims:*** Histidine triad nucleotide-binding protein 1 (HINT1) exhibits proapoptotic and tumor-suppressive activity. HINT1 binds to transcription factors such as teneurin1 and to the regulator of G protein signaling 17 (RGS) (Z2) protein, which incorporates the small ubiquitin-like modifier (SUMO), and is implicated in several types of cancer. HINT1 interacts with proteins such as PKCγ and Raf-1 through zinc ions provided by the cysteine-rich domain of RGSZ2 and the coupled neural nitric oxide synthase (nNOS). Recently, a series of HINT1 mutants have been reported to cause human autosomal recessive axonal neuropathy with neuromyotonia (ARAN-NM). However, the specific alteration in the function of HINT1 induced by these mutants remains to be elucidated. Because sumoylation modifies protein association and transcriptional regulation, we investigated whether HINT1 exhibits zinc- and redox-regulated sumoylase activity, which may be altered in those mutants.

***Results:*** HINT1 exhibits cysteine protease activity to remove SUMO from a variety of signaling proteins. HINT1 sumoylase activity is blocked by zinc, and it is released by nitric oxide or calcium-activated calmodulin (CaM). HINT1 contains a SUMO-interacting motif (110–116 HIHLHVL) and the catalytic triad Cys84-Asp87-His114 in the C-terminal region. Thus, zinc probably provided by the RGSZ2–nNOS complex may bind to Cys84 to block HINT1 isopeptidase activity.

***Innovation:*** To date, HINT1 is the only sumoylase that is regulated by two alternate pathways, redox- and calcium-activated CaM.

***Conclusion:*** The 15 human HINT1 mutants reported to cause ARAN-NM exhibited altered sumoylase activity, which may contribute to the onset of this human motor disease.

## Introduction

The histidine triad nucleotide-binding protein 1 (HINT1) is a zinc-binding protein of ∼14 kDa that is highly conserved in phylogeny. HINT1 is widely expressed in the central nervous system (CNS) and other tissues (27, 32), and at the cellular level, this protein is present in the plasma membrane, nucleus, and cytoplasm. HINT1 was initially described as a protein kinase C (PKC)-inhibiting protein (39), and, indeed, conventional PKCγ and PKCα establish nitric oxide (NO) and zinc-dependent inhibitory associations with HINT1 (43, 44). Later, crystallization studies indicated that HINT1 exists as a homodimer with the protomers interacting through their C-terminal sequences (26, 35), and its amino acid sequence revealed that it belongs to the histidine triad (HIT) family with HINT2 and HINT3 as its closest paralogs. In *in vitro* assays, the HINT protein family exhibits phosphoramidase activity for adenosine-5′-*O*-monophosphoramidate and acts as efficient aminoacyl-adenylate hydrolases (8, 11). In addition, HINT1 catalyzes lysyl-adenylate generated by lysyl-tRNA synthetase and the desulfuration of 5′-*O*-phosphorothioylated nucleosides (22, 38).

**Innovation**

Histidine triad nucleotide-binding protein 1 (HINT1) exhibits zinc-regulated cysteine protease activity to desumoylate a variety of substrates. In addition, in contrast to its adenylate hydrolase activity, this novel function is not shared by HINT2 and HINT3 proteins. HINT1 is the only sumoylase regulated by redox and calmodulin signaling. Similar to the second family of sumoylases, desumoylating isopeptidases, HINT1 has a dimeric structure, forms stable complexes with substrates, and exhibits poor endopeptidase activity toward small ubiquitin-like modifier precursor forms. This activity of HINT1 may be essential for its antitumor activity and regulation of transcription factors. Thus, HINT1 dysfunction may contribute to different types of cancer and to axonal neuropathies with neuromyotonia.

Recently, interest in this protein has increased considerably because an initial report described a series of human HINT1 mutants as the cause of the devastating condition autosomal recessive axonal neuropathy with neuromyotonia (ARAN-NM) (73). Actually, 15 HINT1 mutants have been implicated in the pathophysiology of this neural dysfunction (34, 40, 62). Evidently, the identification of HINT1 features, which may be altered in these human mutants, is of outstanding interest. Thus, a few of the HINT1 mutants exhibit anomalies in their hydrolase lysyl-adenylate activity or in their capacity to constitute the dimeric form (54), whereas other HINT1 mutants still display normal enzymatic activity, which is independent of HINT1 proapoptotic activity (66). These data suggest another not-yet-discovered HINT1 function, which could be altered in the ARAN-NM mutants.

The current literature describes the enzymatic activity of HINT1 mentioned earlier and that zinc- and redox-dependent and -independent processes weave together to enable HINT1 to interact with a series of signaling proteins in the CNS. HINT1 interacts with G protein-coupled receptors (GPCRs) (18) and inotropic glutamate *N*-methyl-d-aspartate receptors (NMDARs) (42, 44). HINT1 binds simultaneously to the cytosolic C terminus of the mu-opioid receptor (MOR) (18) and to regulators of G protein signaling (RGS) proteins of the Rz family, such as RGSZ1 and RGSZ2 (1, 45). In the ternary complex GPCR-HINT1-RGSZ2, HINT1 signaling is regulated by zinc and redox processes. RGSZ2, which carries a zinc-binding cysteine-rich domain (CRD) at their N-terminal sequence (50), is associated with the N-terminal PDZ domain of neural nitric oxide synthase (nNOS) (14). MOR or NMDAR activation promotes nNOS production of NO to remove zinc ions from RGSZ2 CRD (48, 50). Subsequently, conventional PKCs, such as PKCα and PKCγ, or Raf-1, bind through their respective CRDs to HINT1 in a zinc-dependent manner (44, 47).

HINT1 couples with the cleaved N-terminal intracellular domain (ICD) of transmembrane protein teneurin1 (51), and, in the nucleus, this complex induces the activity of the microphthalmia-associated transcription factor (51). HINT1 also inhibits the transcription of target genes (10) and forms a stable association with the Pontin/Reptin complex to inhibit the β-catenin transcriptional pathway (60, 65). Therefore, HINT1 plays a role as a transcriptional repressor. In this context, HINT1 is recruited by the DNA damage response (21, 24), triggers apoptosis (66), exhibits tumor-suppressive activity (25, 57, 70), and inhibits proliferation in human gastric and colon cancer cells (61, 64).

In searching for the unknown activity of HINT1, we noticed that a series of HINT1-interacting proteins, such as transcription factors and RGS-Rz proteins, which have been implicated in multiple human cancers (7, 19), are regulated by covalent conjugation of the small ubiquitin-like modifier (SUMO) (16, 41). Notably, sumoylated RGSZ1 and RGSZ2 proteins are found not only at the neural membrane but also in the nucleus (41), where, as reported for ICD teneurin1, they interact with HINT1 to participate in transcriptional processes. As HINT1 interactions with signaling proteins and nucleotide hydrolase activity can be regulated by zinc (43, 53), we studied whether HINT1 might regulate SUMO post-translational modification of interacting proteins by zinc and redox mechanisms and whether such a novel function was found to be altered in the ARAN-NM-related mutants. SUMO proteases are cysteine proteases with at least one histidine in the catalytic site (20), and HINT1 fulfills this criterion because it carries two cysteines and seven histidines in its sequence.

Our study shows that HINT1 cleaves sumoylated substrates, such as RGS-Rz proteins, ICD teneurin1, and Ran GTPase activating protein 1 (RanGAP1), and that this activity is inhibited by zinc and promoted by NO or calcium-activated calmodulin (CaM). Notably, isopeptidase activity was altered in the fifteen human HINT1 mutants reported thus far.

## Results

**FIG. 1.**
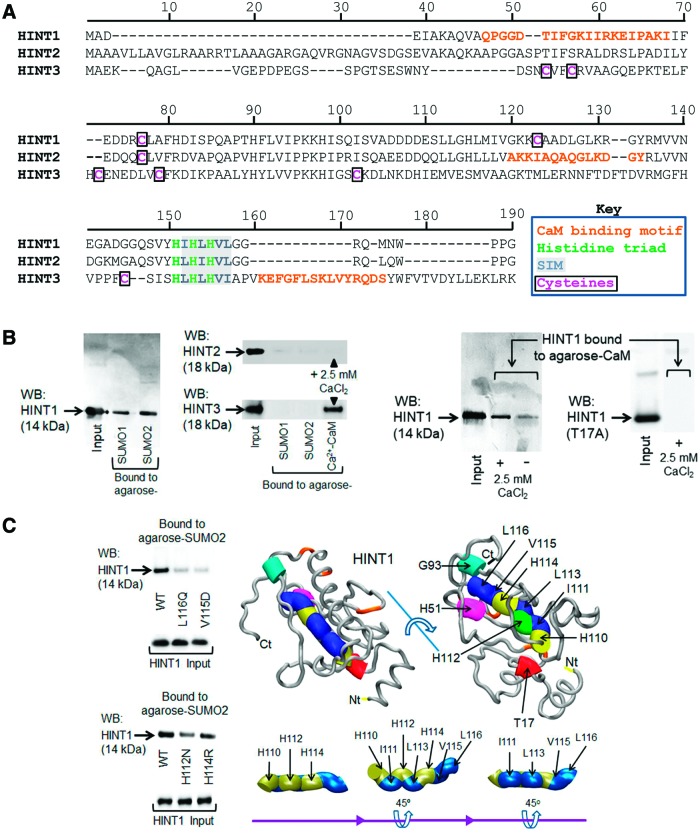
**HINT1 binds to SUMO proteins and calcium-activated CaM**. **(A)** Sequence alignment of the three HINT proteins showing their limited similarity to the HIT linear sequence. Each HINT protein contains a putative CaM-binding motif but with a different amino acid sequence. **(B)** HINT1 but not HINT2 or HINT3 binds SUMO proteins. HINT1 and HINT3 but not HINT2 bind CaM in the presence of 2.5 m*M* CaCl_2_. **(C)** HINT1 linear sequence 110–116 contains the HIT and the SIM, which form opposing surfaces. The HINT1 SIM mutants studied weakly bind to SUMO. The HINT1 3D structure is shown as a tube with reference amino acids as colored cylinders (tube occupancy). The HINT1 protein and the SIM-His triad region are rotated to show their 3D organization. The HINT structural models shown herein were predicted by Novafold (DNASTAR, Inc., Madison, WI). Details of immunoblot detection in “Materials and Methods” section and Supplementary Figure S4. 3D, three-dimensional; CaM, calmodulin; HINT1, histidine triad nucleotide-binding protein 1; HIT, histidine triad; SIM, sumo-interacting motif; SUMO, small ubiquitin-like modifier. Color images are available online.

In humans, the HINT family includes three members, HINT1 with 126 amino acids, HINT2 with 163 amino acids, and HINT3 with 165 amino acids (Fig. 1A). Close to their C terminus, these proteins contain the conserved HIT, which alternates with hydrophobic amino acids HI(L)HL(I)HVL(I) in a typical setup of a SUMO-interacting motif (SIM) (71). In *in vitro* assays, we observed that HINT1, but not HINT2 or HINT3, bound to SUMO1 and SUMO2 proteins. Moreover, protein analysis indicated the presence of a CaM-binding motif but in a different amino acid sequence for each of the HINT proteins (68). Thus, HINT1 and HINT3 exhibited binding to CaM, and in the presence of physiological levels of calcium (2.5 m*M*), the HINT1–CaM association was increased (Fig. 1B). Accordingly, the T17A mutation inside the predicted HINT1 CaM binding motif (12–31 QPGGD**T**IFGKIIRKEIPAKI) abrogated Ca^2+^-CaM binding. On the other hand, SUMO2–HINT1 association was greatly reduced after altering the predicted SIM amino acids, that is, in V115D and L116Q and to a lesser extent in the HINT1 mutants of the accompanying H112N and H114R (Fig. 1C) [*F*(4,15) = 169.175, *p* < 0.001; V115D *t* = 22.02, *p* < 0.001; L116Q *t* = 19.14, *p* < 0.001; H112N *t* = 13.37, *p* < 0.001; H114R *t* = 5.59, *p* < 0.01].

**FIG. 2.**
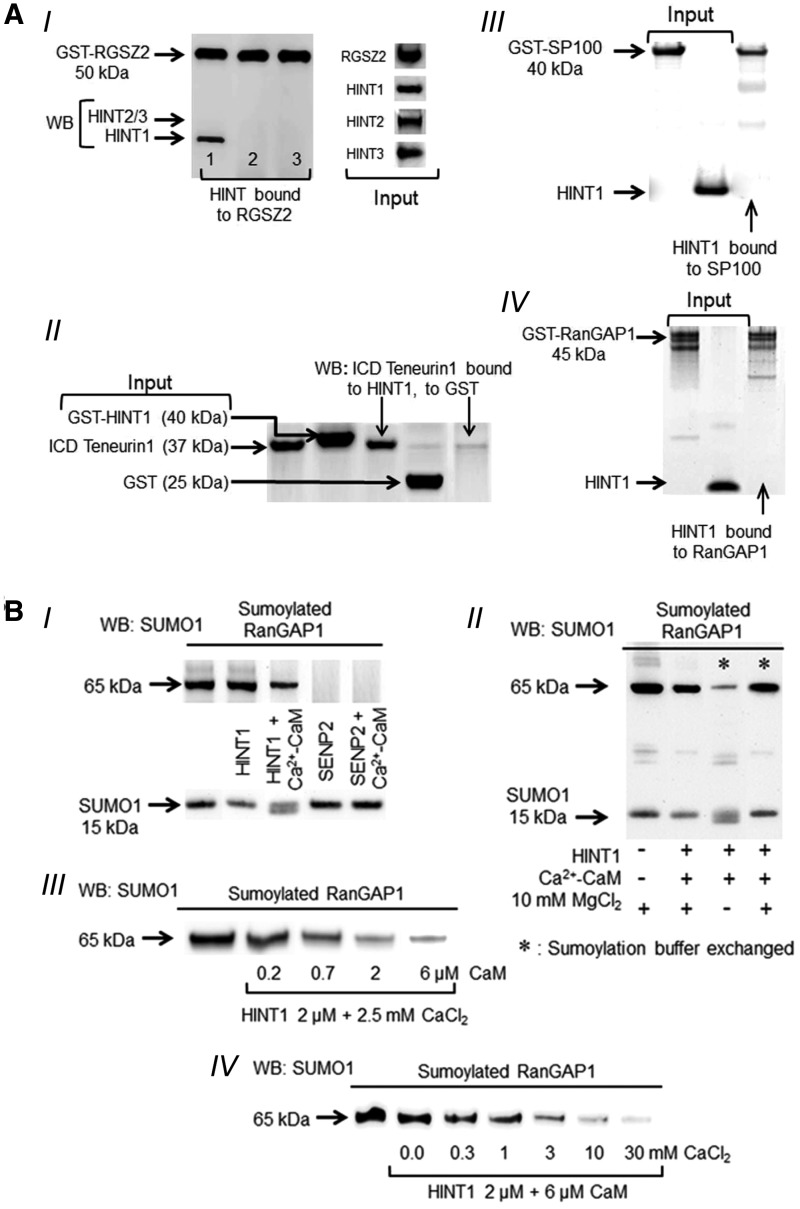
**HINT1 exhibits isopeptidase activity**. **(A)** HINT1 interactions with the proteins used in the study. **(I)** HINT1 but not its paralogs forms stable complexes with RGSZ2 proteins. **(II)** ICD teneurin1 interacts with HINT1 but not with GST. **(III**, **IV)** HINT1 associations with RanGAP1 and SP100 were weak or not detected. **(B)** HINT1 exhibits SUMO protease activity on sumoylated RanGAP1, which is regulated by calcium and CaM. **(I)** HINT1 (2 μ*M*) but not SENP2 (0.3 μ*M*) requires calcium-activated CaM (6 μ*M*) to exhibit sumoylase activity. **(II)** Removal of 10 m*M* MgCl_2_ from the sumoylation buffer greatly improves Ca^2+^-CaM-dependent HINT1 isopeptidase activity on sumoylated RanGAP1. **(III**, **IV)** HINT1 exhibits isopeptidase activity on sumoylated RanGAP1 in the presence of increasing concentrations of CaCl_2_ and of CaM. Details of immunoblot detection in “Materials and Methods” section and Supplementary Figures S5 and S6. GST, glutathione *S*-transferase; ICD, intracellular domain; RanGAP1, Ran GTPase-activating protein 1; RGSZ2, regulator of G protein signaling 17 (Z2); SENP, sentrin-specific protease.

In *in vitro* assays, HINT1 formed stable complexes with ICD teneurin1 and HINT1, but not HINT2 or HINT3, associated with the RGSZ2 protein, with 2.5 m*M* calcium promoting and Ca^2+^-CaM diminishing these associations (Fig. 2A[I, II], Supplementary Fig. S1A, B). Stable interactions were not observed for HINT1 with glutathione *S*-transferase (GST) or with proteins frequently used in *in vitro* sumoylation assays such as SP100 and a RanGAP1 fragment (Fig. 2A[III, IV]). Initially, we addressed the isopeptidase activity of HINT1 on sumoylated RanGAP1 in the presence of the sumoylation mix. Although SUMO/sentrin-specific protease 2 (SENP2) efficaciously removed SUMO1 from sumoylated RanGAP1, the HINT1 protein achieved only partial removal of SUMO1, and their activity was similar in the absence or presence of additional Ca^2+^-CaM (Fig. 2B[I]). Because the sumoylation buffer contained 10 m*M* MgCl_2_, and Mg^2+^ may interfere with calcium activation of CaM (15), the activity of HINT1 was addressed in the absence of Mg^2+^ (buffer exchange). Under these conditions, HINT1 efficaciously removed SUMO1 from RanGAP1 (Fig. 2B[II]), and the isopeptidase activity of 2 μ*M* HINT1 was maximal in the presence of 6 μ*M* CaM and ∼10 m*M* CaCl_2_ (Fig. 2B[III, IV]).

**FIG. 3.**
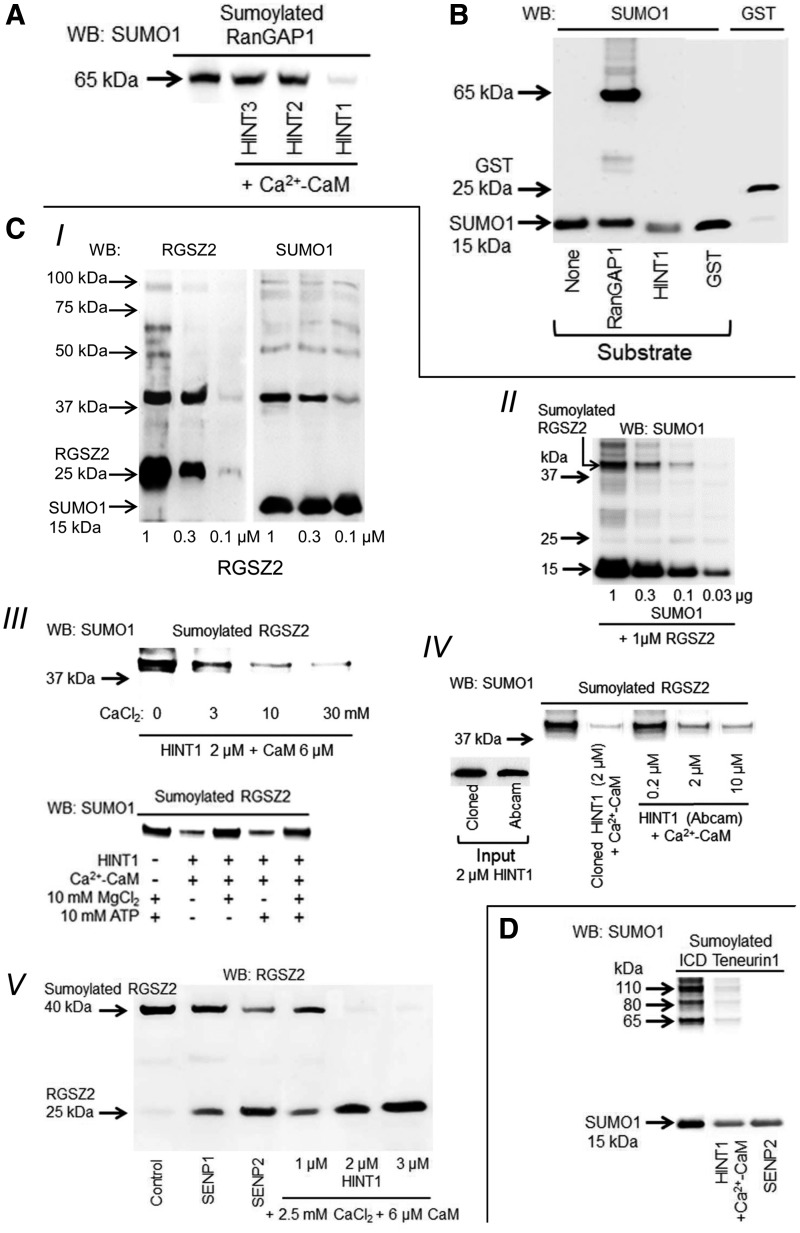
**HINT1 removes SUMO1 from sumoylated RanGAP1, RGSZ2, and ICD teneurin1. (A)** HINT2 and HINT3 do not exhibit Ca^2+^-CaM-dependent sumoylase activity. **(B)** In the RanGAP1 sumoylation assay, HINT1 and GST did not incorporate SUMO. **(C[I**, **II])** The RGSZ2 protein incorporated SUMO1. **(III)** HINT1 cleaved SUMO from the RGSZ2 protein in a calcium- and CaM-dependent manner. HINT1 isopeptidase activity on sumoylated RGSZ2 greatly diminished in the presence of 10 m*M* MgCl_2_ but not 10 m*M* ATP. **(IV)** HINT1 from a different source (Abcam; #ab87362) exhibited sumoylase activity on RGSZ2 similar to that observed with our cloned HINT1. **(V)** HINT1, SENP1, and SENP2 desumoylated and preserved RGSZ2 size. **(D)** ICD teneurin1 showed various putative sites for sumoylation (71). HINT1 and SENP2 desumoylated ICD teneurin1. Further details of immunoblot detection in “Materials and Methods” section and Supplementary Figures S6 and S7.

In the following assays, the sumoylase activity of HINT1 was determined while maintaining the parameters mentioned earlier. In these experimental conditions, HINT2 and HINT3 did not exhibit sumoylase activity (Fig. 3A). In the *in vitro* sumoylation assay, HINT1 and GST did not incorporate SUMO1 (Fig. 3B). HINT1 also displayed isopeptidase activity to remove SUMO from its interacting proteins RGSZ2 and ICD teneurin1. The sumoylation of RGSZ2 was SUMO1 dependent and provided a main band of ∼40 kDa (Fig. 3C[I, II]). In the presence of Ca^2+^-CaM, HINT1 efficaciously removed SUMO1 from the RGSZ2 protein, whereas 10 m*M* MgCl_2_ but not 10 m*M* ATP diminished its isopeptidase activity (Fig. 3C[III]). We observed that recombinant HINT1 obtained from a commercial source (Abcam plc, Cambridge, United Kingdom; #ab87362) also removed SUMO1 from sumoylated RGSZ2 (Fig. 3C[IV]). Desumoylation of RGSZ2 by HINT1 and SENP1/2 preserved the native protein, which was now detected at 25 kDa (Fig. 3C[V]). HINT1 and SENP2 also removed SUMO1 from ICD teneurin1 (Fig. 3D). In contrast to the result observed for SENP2, HINT1 barely desumoylated SP100 or cleaved polymeric SUMO2/3 chains and lacked SUMO-processing activity (Supplementary Fig. S2A–C).

**FIG. 4.**
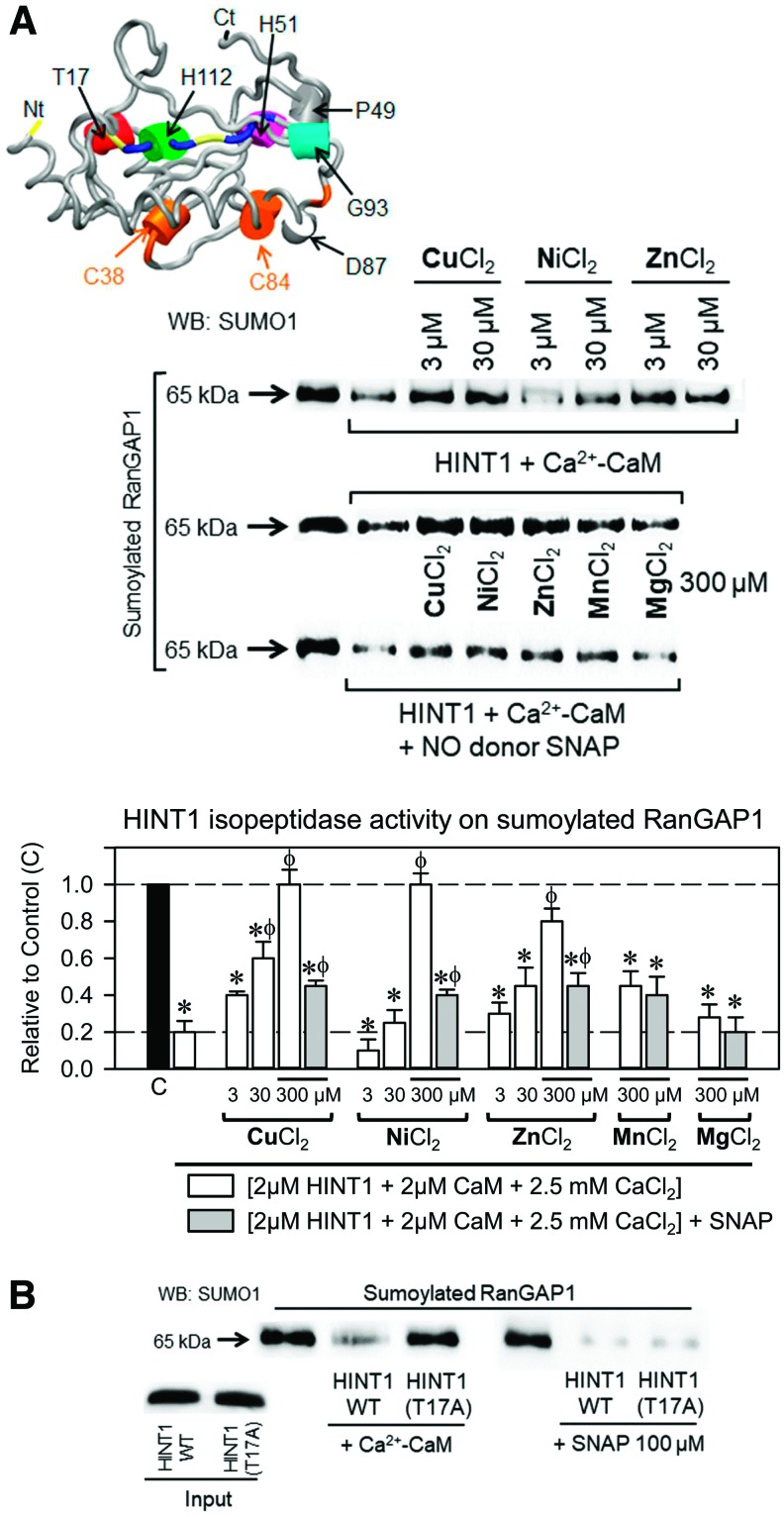
**Effect of divalent metal ions on HINT1 and SENP2 isopeptidase activity and identification of the HINT1 CaM-binding motif. (A)** Concentrations of 3, 30, and 300 μ*M* chloride salts of Cu, Ni, Zn, Mn, and Mg divalent metal cations were added to HINT1 (2 μ*M*) desumoylation buffer of sumoylated-RanGAP1. The concentration of 300 μ*M* of the aforementioned salts was also studied in the presence of the NO donor SNAP (100 μ*M*). Each bar is the computed mean ± standard error of the mean of three determinations. Data refer to the control group, which did not include HINT1 and was assigned an arbitrary value of 1; “*” significantly different compared with the control group, “ϕ” indicates a significant difference from the group that received HINT1 but not the metal ion. ANOVA, Holm-Sidak multiple comparisons, *p* < 0.05. **(B)** The predicted CaM-binding site in the HINT1 protein is located in its N-terminal region. In the presence or absence of Ca^2+^-CaM, the HINT1 T17A mutant did not exhibit isopeptidase activity; however, the NO donor SNAP (100 μ*M*) rescued T17A HINT1 sumoylase activity on RanGAP1, which was comparable to that of the WT. Further details of NO donors in Supplementary Figure S1C. ANOVA, analysis of variance; NO, nitric oxide; SNAP, *S*-nitroso-*N*-acetyl-dl-penicillamine; WT, wild type. Color images are available online.

The purine nucleoside phosphoramidase and adenylate hydrolase activity of HINT1 is reduced by a series of divalent cations (53), and Ca^2+^-CaM-activated HINT1 isopeptidase activity is also diminished in the presence of Cu^2+^, Zn^2+^, and Ni^2+^ chloride salts. Although 10 m*M* Mg^2+^ abrogated the effect of Ca^2+^-CaM (Fig. 2B[II]), Mg^2+^ and Mn^2+^ up to 300 μ*M* had no effect in this paradigm (Fig. 4A). Cysteines and histidines are the amino acids that exhibit the greatest affinity for divalent metal cations such as Cu^2+^, Zn^2+^, and Ni^2+^ (72), and NO disrupts their binding to cysteine thiol groups but not to histidines (29). In the presence of *S*-nitroso-*N*-acetyl-dl-penicillamine (SNAP), an NO donor, the capacity of Cu^2+^, Zn^2+^, and Ni^2+^ to antagonize HINT1 sumoylase activity diminished. These observations suggested that HINT1 sumoylase activity was promoted by NO. Indeed, Ca^2+^-CaM failed to activate SUMO protease activity in the HINT1 T17A mutant, which exhibited this activity in the presence of SNAP (Fig. 4B). Other NO donors, such as (2*E*,3*E*)-4-ethyl-2-(hydroxyimino)-5-nitro-3-hexeneamide (NOR-3) and spermine NONOate, also promoted HINT1 sumoylase activity and this activity was observed even in the presence of 10 m*M* MgCl_2_ (Supplementary Fig. S1C).

**FIG. 5.**
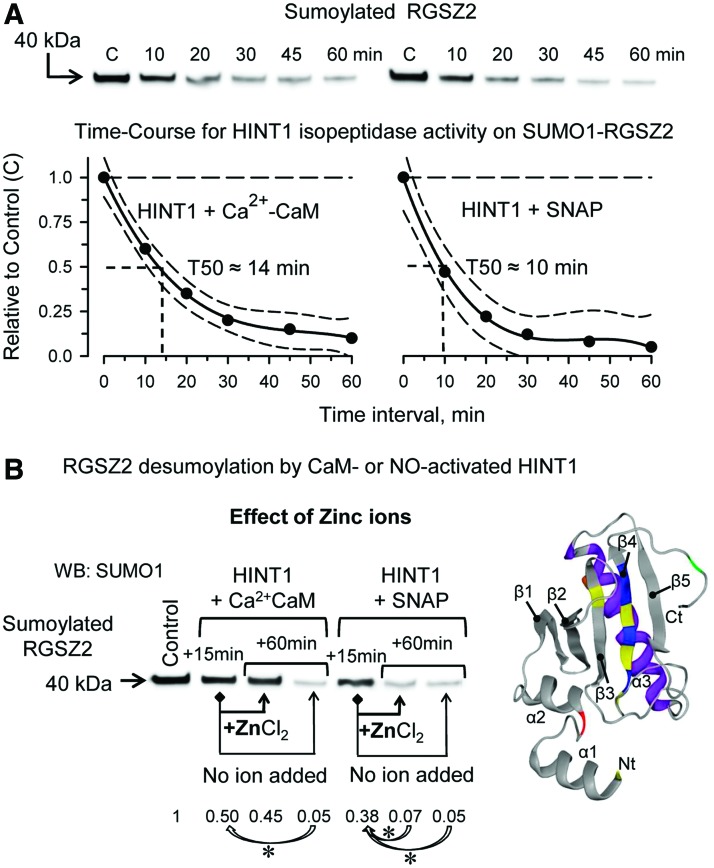
**HINT1 isopeptidase activity, time-course and effect of zinc ions. (A)** Time-course for HINT1 activated by Ca^2+^-CaM to remove SUMO1 from the RGSZ2 protein. The data were analyzed by nonlinear regression (Sigmaplot/Sigmastat v 14.0; Systat Software, Inc.), (estimate ± SE): *r* = 0.97 ± 0.04, T50 = 12.35 ± 0.27 min, *t* = 37.21, DF total = 6, MS = 0.26, *p* < 0.05. Identical assay, but HINT1 was activated by the NO-donor SNAP (estimate ± SE): *r* = 0.99 ± 0.07, T50 = 6.31 ± 0.23 min, *t* = 57.79, DF total = 6, MS = 0.215, *p* < 0.05. “C” denotes control group without HINT1 isopeptidase activator. The curve fits and 95% confidence intervals are shown. **(B)** Zinc ions added during the time-course blocked HINT1 isopeptidase activity activated by Ca^2+^-CaM, but failed when NO was the activator. HINT1 (2 μ*M*), SNAP (100 μ*M*), CaCl_2_ (2.5 m*M*), CaM (6 μ*M*), sumoylated RGSZ2 (1 μ*M*), and ZnCl_2_ (300 μ*M*). Data refer to the control group, which did not include HINT1 and was assigned an arbitrary value of 1; “*” for each group, Ca^2+^-CaM or SNAP indicates a significant difference with respect to the +15-min time interval. ANOVA, Holm-Sidak multiple comparisons, *p* < 0.05. Further details of immunoblot detection in “Materials and Methods” section and Supplementary Figure S8. DF, degrees of freedom; MS, mean square; SE, standard error. Color images are available online.

**FIG. 6.**
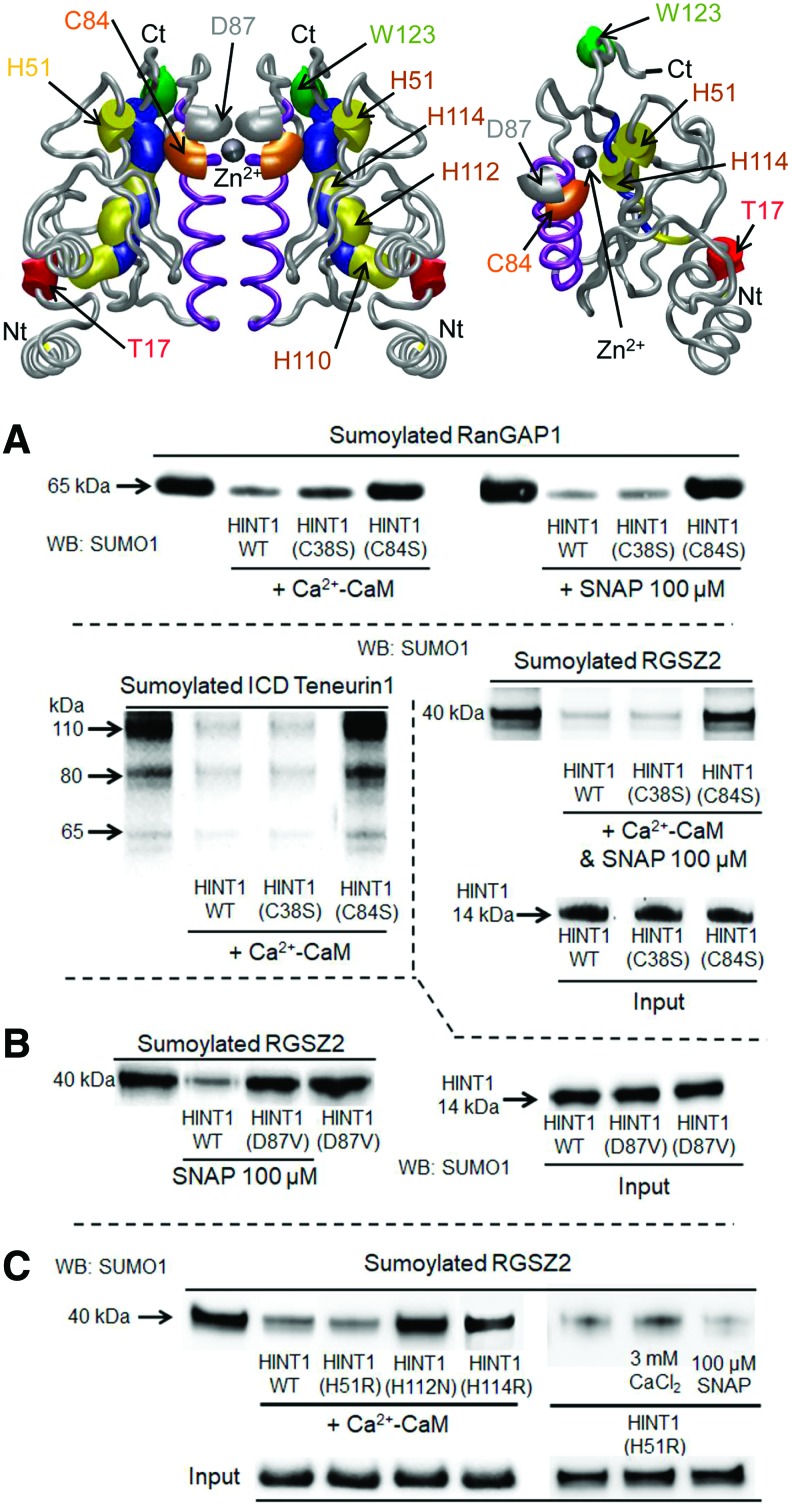
**Identification of HINT1 isopeptidase catalytic domain. (A)** Identification of the cysteine residue implicated in isopeptidase activity. In the presence of Ca^2+^-CaM, SNAP, or both, the HINT1 C38S mutant but not the C84S cleaved SUMO from GST-RanGAP1, RGSZ2, and ICD teneurin1. **(B)** The HINT1 D87V mutant did not exhibit isopeptidase activity in the presence or absence of SNAP. **(C)** In the presence of Ca^2+^-CaM or SNAP, HINT1 mutants H112N and H114R did not exhibit isopeptidase activity on sumoylated RGSZ2, but H51R was active even in the absence of these activators. HINT1 (2 μ*M*), CaCl_2_ (2.5 m*M*), CaM (6 μ*M*), SNAP (100 μ*M*), sumoylated RGSZ2, ICD teneurin1, and RanGAP1 (1 μ*M*). Further details of immunoblot detection in “Materials and Methods” section and Supplementary Figures S6 and S7. Color images are available online.

HINT1 removed SUMO from RGSZ2 proteins with a T50 of ∼10–15 min and was slightly faster when SNAP, instead of Ca^2+^-CaM, was the activator (Fig. 5A). The addition of zinc ions interrupted the Ca^2+^-CaM-activated isopeptidase activity of HINT1 but failed when SNAP/NO promoted this activity (Fig. 5B). These observations strongly suggested that HINT1 isopeptidase activity is inhibited by divalent metal cations, such as zinc, and, indeed, the metal chelator *N*,*N*,*N*′,*N*′-tetrakis(2-pyridylmethyl) ethylenediamine (TPEN) promoted HINT1 desumoylase activity in the absence of Ca^2+^-CaM or SNAP (Supplementary Fig. S1D). In the presence of Ca^2+^-CaM or SNAP, the C38S HINT1 mutant exhibited SUMO protease activity on sumoylated RanGAP1, RGSZ2, or ICD teneurin1; however, this activity was not observed in the C84S HINT1 mutant (Fig. 6A). The HINT1 mutant D87V was devoid of spontaneous or SNAP-activated isopeptidase activity, suggesting its participation in the catalytic triad (Fig. 6B). Although the HINT1 human mutant H51R exhibited spontaneous desumoylase activity on sumoylated RGSZ2, other HINT1 human histidine mutants, such as H112N and H114R, were devoid of isopeptidase function (Fig. 6C). Inappropriate storage of recombinant HINT1 favored the formation of Cys84-dependent dimers and trimers, which were disrupted by reducing agents such as β-mercaptoethanol. These interactions later become resilient to reduction and negatively affect the capacity of HINT1 to remove SUMO (Supplementary Fig. S3A, B).

**FIG. 7.**
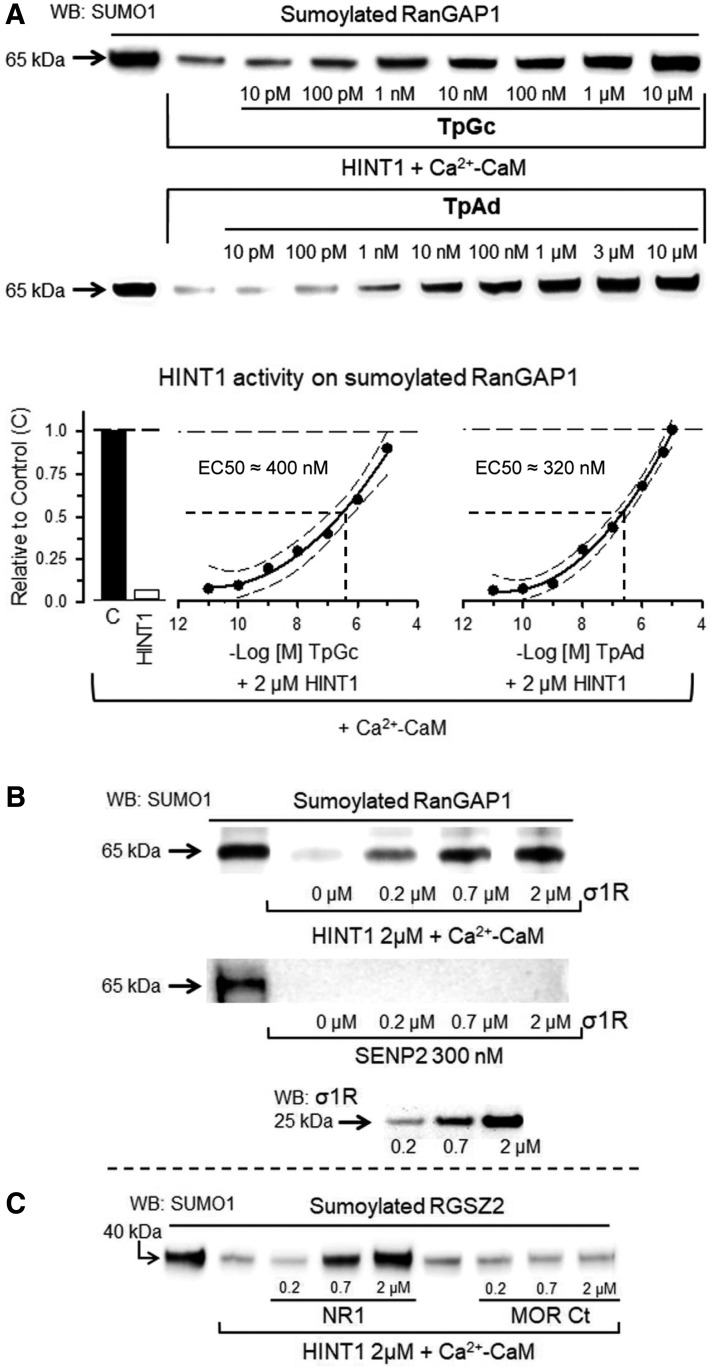
**Regulation of HINT1 sumoylase activity by drugs affecting its adenylate hydrolase activity and third-partner interacting proteins. (A)** The substrate TpAd, and the inhibitor TpGc of HINT1 adenylate hydrolase activity reduced the capacity of HINT1 to remove SUMO from RanGAP1 in a dose-dependent manner. The data were analyzed by nonlinear regression, competition at a single site (Sigmaplot/Sigmastat v 14.0; Systat Software, Inc.) and shown as curve fit and 95% confidence interval. TpGc (estimate ± SE): *r* = 0.93 ± 0.12, EC50 = 403.18 ± 12.20 n*M*, *t* = 16.13, DF total = 7, MS = 0.22, *p* < 0.05; TpAd (estimate ± SE): *r* = 0.97 ± 0.11, EC50 = 320.78 ± 15.85 n*M*, *t* = 19.31, DF total = 7, MS = 0.39, *p* < 0.05. **(B)** Effect of HINT1-interacting signaling proteins on HINT1 sumoylase activity: The σ1R long isoform reduced the sumoylase activity of HINT1 but not SENP2 in a concentration-dependent fashion. **(C)** The cytosolic C-terminal region C0–C1–C2 of the NMDAR NR1 subunit, but not the C-terminal region of MOR, reduced HINT1 isopeptidase activity. Further details of immunoblot detection in “Materials and Methods” section. MOR, mu-opioid receptor; NMDAR, *N*-methyl-d-aspartate receptor; TpAd, phosphoramidate tryptamine AMP; TpGc, guanosine-5′-tryptamine carbamate.

As mentioned earlier, HINT1 displays nucleoside phosphoramidase and acyl-AMP hydrolase activity in *in vitro* assays. This HINT1 enzymatic activity can be promoted by phosphoramidate tryptamine AMP (TpAd) and inhibited by guanosine-5′-tryptamine carbamate (TpGc) (3, 4). Notably, in our desumoylation assay, TpAd and TpGc reduced the activity of HINT1 in a concentration-dependent manner with apparent ED50s of 320 and 403 n*M*, respectively (Fig. 7A), suggesting that HINT1 enzymatic activities may share critical amino acid residues at the catalytic site and HIT/SIM domain.

**FIG. 8.**
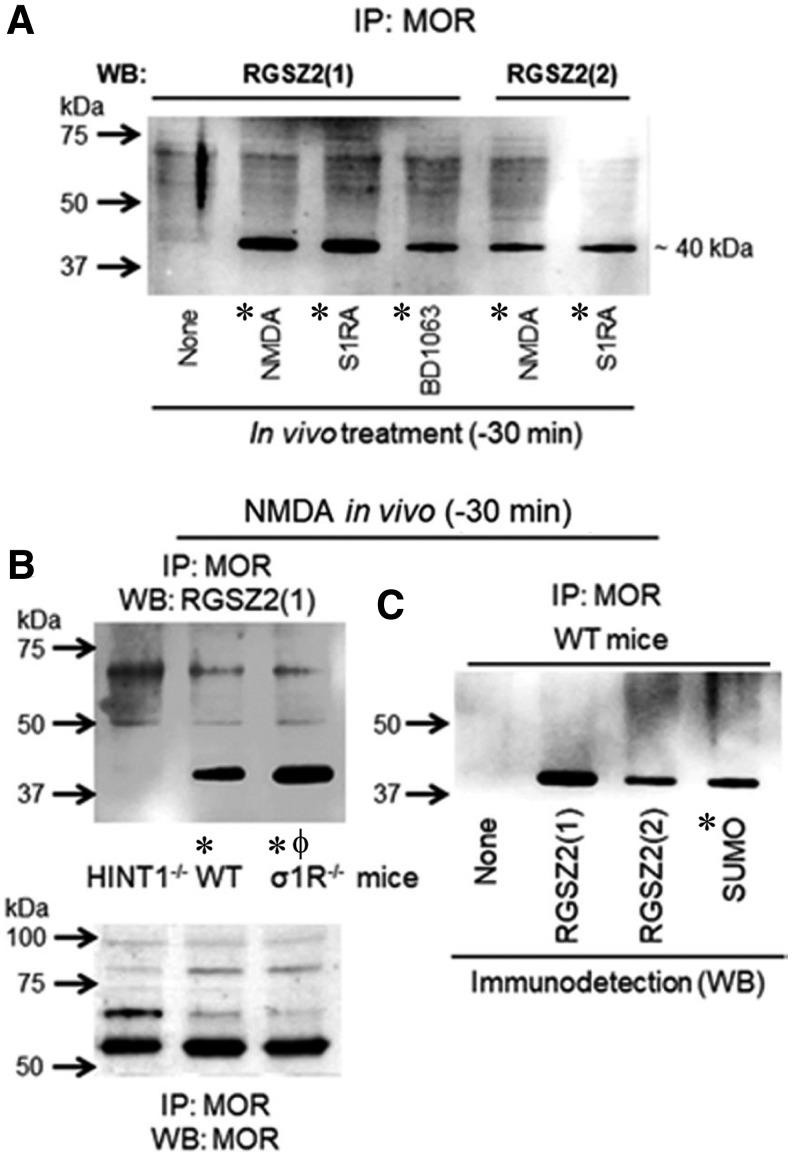
***In vivo* regulation of σ1R and glutamate NMDAR promotes HINT1 binding to sumoylated RGSZ2 proteins. (A)** The icv administration of NMDA, a glutamate NMDAR agonist, or of S1RA and BD1063, σ1R antagonists, greatly enhanced the coprecipitation of RGSZ2 proteins with MOR–HINT1 complexes. The mice were sacrificed 30 min after the icv-injection of the drugs, and *ex vivo* determinations were performed in cortex synaptosomes. IP, immunoprecipitation; WB, Western blot; carried out with two antibodies, RGSZ2(1) and RGSZ2(2), directed against distinct domains of the target protein. “*” Significantly different from the control group (stated as None). **(B)** The *in vivo* administration of NMDA to WT and σ1R^−/−^ mice caused the association of sumoylated RGSZ2 with MOR but failed in HINT1^−/−^ mice, “*” significantly different from the HINT1^−/−^ group; “*ϕ*” significantly different from the WT group. **(C)** In NMDA-treated mice, the RGSZ2 associated with MOR-HINT1 was sumoylated, “*” significantly different from the control group (stated as None). **(A**–**C)** The assays were repeated at least twice. ANOVA, Holm-Sidak multiple comparisons, *p* < 0.05. Further details of immunoblot detection in “Materials and Methods” section and Supplementary Figure S9. icv, intracerebroventricular; NMDA, *N*-methyl-d-aspartate.

Several HINT1 interactions with third-partner proteins depend on zinc-bound cysteines, which are regulated by redox processes (43). In this study, σ1R diminished the sumoylase activity of HINT1. However, σ1R did not alter SENP2 activity (Fig. 7B). The cytosolic C-terminal sequence of NR1 abolished HINT1 isopeptidase function, but the cytosolic C-terminal region of MOR did not (Fig. 7C). Next, we explored the possible significance of this regulation of HINT1 isopeptidase activity *in vivo*. Mice were intracerebroventricularly injected with *N*-methyl-d-aspartate (NMDA), a glutamate NMDAR agonist, and the σ1R antagonists S1RA and BD1063. These procedures diminish the *in vivo* association of NMDAR NR1 subunits and of σ1Rs with MOR–HINT1 complexes (42, 44). After 30 min, we analyzed the coprecipitation of the RGSZ2 protein with the MOR *ex vivo*. This association was barely detected in control mice but greatly increased in response to NMDA and σ1R antagonists (Fig. 8A). Although NMDA promoted MOR–RGSZ2 associations in wild type (WT) mice, the effect was greater in σ1R^−/−^ mice and absent in HINT1^−/−^ mice (Fig. 8B). The RGSZ2 coimmunoprecipitated with the MOR was sumoylated (Fig. 8C). Thus, *in vivo*, NMDARs and σ1Rs negatively regulate the interaction of HINT1 proteins with sumoylated RGSZ2 proteins and probably its isopeptidase activity as well.

**FIG. 9.**
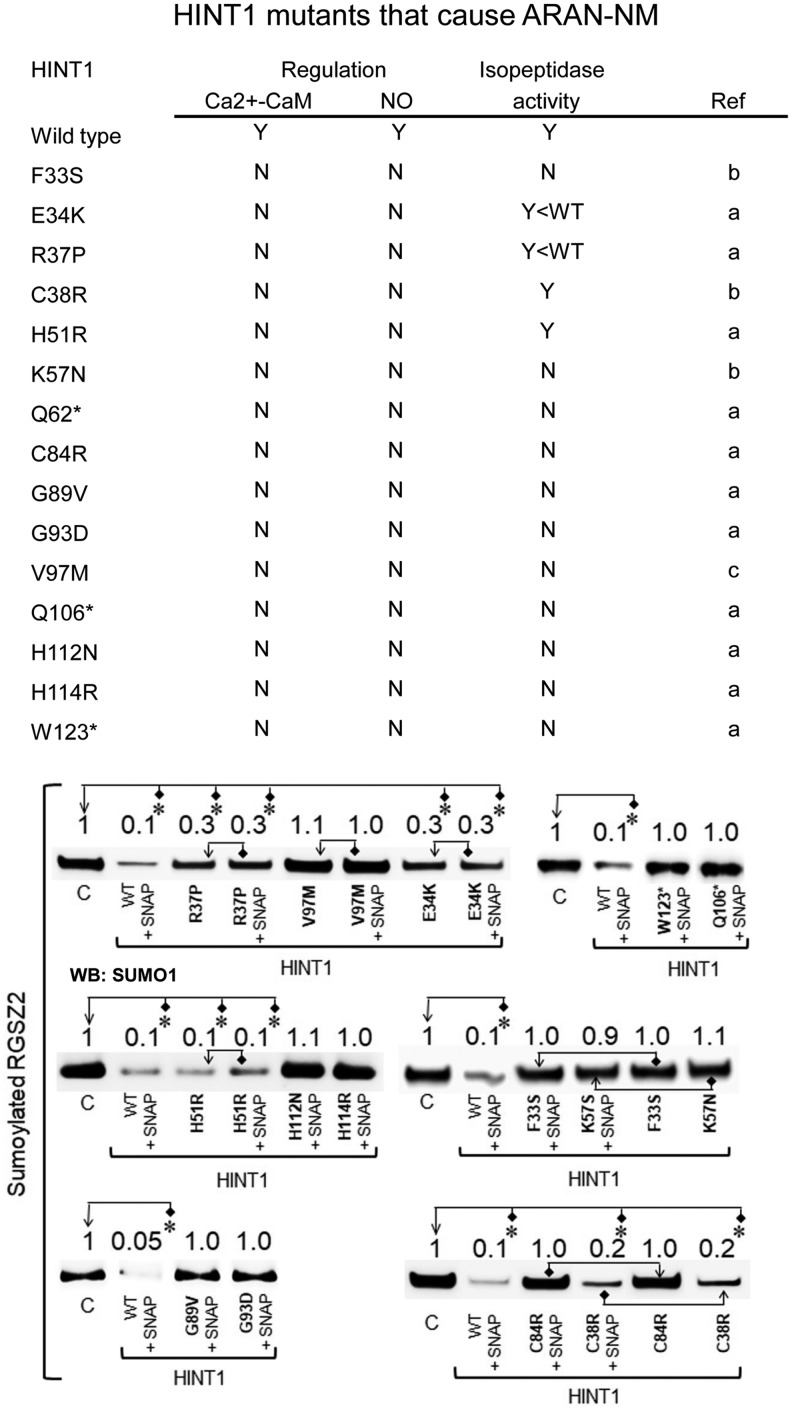
**Human HINT1 mutants that cause ARAN-NM exhibit impaired isopeptidase activity.** The NO donor SNAP failed to activate the sumoylase activity in most of the human HINT1 mutants evaluated. The E34K, R37P, and H51R mutants showed deregulated spontaneous activity to remove SUMO1 from RGSZ2 proteins. Identical results were obtained by using Ca^2+^-CaM instead of SNAP (not shown). The HINT1 mutant Q62* in which the catalytic site is absent was assumed to lack isopeptidase activity. The *columns* describing isopeptidase activity and its regulation: N and Y indicate No and Yes, respectively; Y < WT denotes deregulated isopeptidase activity worse than that of the WT. Ref: references reporting the human HINT1 mutants: a (34); b (40); c (62). The assays were performed at least twice, and each point was duplicated. “*” Significant difference with respect to the control group (C, assigned an arbitrary value of 1), which contained the sumoylated RGSZ2 protein but not the HINT1 protein; ANOVA, Holm-Sidak multiple comparisons, *p* < 0.05. SNAP (100 μ*M*). Further details of immunoblot detection in “Materials and Methods” section and Supplementary Figure S10. HINT1 mutants not described in humans but included in the study are in Supplementary Table S1. ARAN-NM, autosomal recessive axonal neuropathy with neuromyotonia.

At the time we performed this study, the number of HINT1 mutants reported to cause ARAN-NM in humans was 15 (34, 40, 62). Thus, we addressed the capacity of these mutants to remove SUMO from sumoylated RGSZ2 and whether this activity was regulated by NO or Ca^2+^-CaM. Most HINT1 human mutants did not exhibit isopeptidase activity and could not be recruited by the activators mentioned earlier. However, C38R and H51R mutants showed deregulated full desumoylase activity, and E34K and R37P also exhibited deregulated but somehow lesser activity than the WT (Fig. 9). Because isopeptidase activity is absent from the cropped Q106* and W123* mutants, we inferred that the Q62* mutant, which lacks the sequence containing the catalytic triad, was also devoid of such enzymatic function.

## Discussion

This study reveals the zinc- and NO-regulated isopeptidase activity of the HINT1 protein, a new feature not shared by the structurally and phylogenetically related HINT2 and HINT3 proteins. The sumoylase activity of HINT1 was triggered by CaM, probably because this calcium-activated protein binds and sequesters inhibitory zinc ions (23, 63) from the HINT1 catalytic site. Thus, the capacity of HINT1 to remove SUMO from membrane and nuclear proteins, such as RGSZ2 and ICD teneurin1, may be of functional relevance to maintain synaptic tonus and to regulate nuclear gene transcription. Indeed, this activity was altered in the human HINT1 mutants that cause ARAN-NM.

Covalent protein sumoylation typically controls the interaction of the modified proteins with other proteins and is reversed by isopeptidases known as sumoylases. The first family of SUMO-specific proteases described was the SENP family, followed by the desumoylating isopeptidase (DeSI) family (58), and then by other isopeptidases related to the Axin-binding protein Axam (37). Sumoylases have the catalytic domain usually located close to the C terminus and contain a conserved His–Asp–Cys triad (5, 12). This triad is present in several cysteine proteases (9) where the cysteine is the nucleophile, the histidine is the base, and the aspartate is the acidic triad member that forms a hydrogen bond with the basic residue. SENPs and DeSIs contain SIM and cysteine-based catalytic sites at their C-terminal region. DeSIs but not SENPs form homodimers with the active site situated in the groove between the two protomers (58). Notably, HINT1 forms dimers, and the protomer contains a SIM at its C-terminal sequence. A series of observations, such as the activation of HINT1 isopeptidase by NO, which is absent in the C84S but not the C38S mutant; the reduction in HINT1 desumoylase function by *in vitro* Cys84-dependent formation of disulfide bridges between protomers; and Cys84 being the only computer-predicted S-nitrosylation site on this protein (67), indicate that HINT1 shares the cysteine protease catalytic organization.

DeSIs harbor only a catalytic dyad with Cys108 and His38, whereas the tridimensional structure of HINT1 suggests the presence of the triad. The HINT1 helical loop facing the dimer interface contains Cys84 close to Asp87, and the required histidine may be provided by His112 or His114, with the latter closer to Cys84 in the HINT1 three-dimensional (3D) structure. SENPs recognize a range of diverse substrates; however, DeSI1 shows sumoylase activity toward only a few substrates (56). In our study, HINT1 exhibited isopeptidase activity on sumoylated RGSZ2, ICD teneurin1, and RanGAP1, but it barely cleaved SUMO from SP100 and very weakly broke polySUMO2/3 chains. Unlike SENPs, HINT1 and DeSIs share their dimeric organization and an extremely low endopeptidase activity toward precursor forms of SUMO1 and SUMO2 (58).

Although the HINT1 sequence does not reveal a “zinc finger” structure, this protein has been identified as a zinc-binding protein (39); however, the capacity of HINT1 to bind calcium remains controversial (32, 33). Among the amino acids, cysteine and histidine display the highest affinity toward metal ions such as Ni^2+^, Zn^2+^, and Cu^2+^. Cysteine residues strongly bind to zinc ions and are the main target of NO signaling through protein modification (29, 72). Thus, NO reacts with cysteine Zn/S sites, promoting the release of zinc and the S-nitrosylation of these thiol groups (29). The nitrosylation of cysteines is readily reversible, typically *via* the S-nitrosoglutathione reductase or thioredoxin systems (55). Because *in vitro* NO does not release zinc from histidines, our results indicate that the HINT1 isopeptidase catalytic site is regulated by a zinc ion bound to a cysteine residue, probably Cys84. This inhibitory switch can be removed by NO and CaM through its calcium-dependent binding to the HINT1 N-terminal sequence (23, 63). However, these HINT1-activating signaling pathways show differences. NO modifies the zinc target on Cys84, whereas CaM removes only the ion without altering its binding site. Thus, free zinc ions may oppose Ca^2+^-CaM but not NO in their activation of HINT1 isopeptidase.

**FIG. 10.**
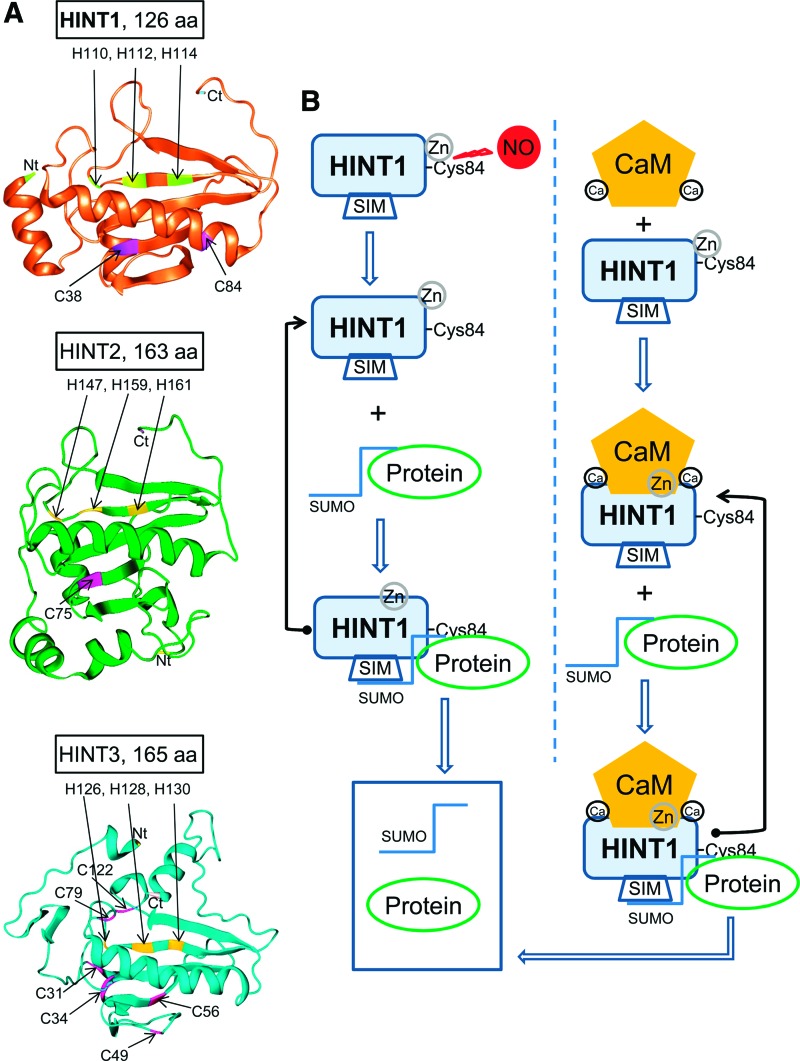
**Dual regulation of HINT1 sumoylase activity. (A)** The HIT nucleotide-binding protein family. Ribbon representation of the 3D structural similarity between HINT1, HINT2, and HINT3 proteins. The HIT is shown in *yellow*, and cysteines are shown in *purple* (Novafold v15; DNASTAR, Inc.). **(B)** NO produces the S-nitrosilation of cysteine thiol groups and activates HINT1 isopeptidase activity. The reduction in cysteine S-nitrosylated thiol groups, typically *via* the S-nitrosoglutathione reductase or thioredoxin systems, enables zinc to bind to HINT1 and inhibit its desumoylase activity. Calcium-activated CaM releases zinc inhibitory control on HINT1 isopeptidase activity. Reductions in calcium levels will cause calcium-free inactive CaM to dissociate from HINT1. In this situation, zinc ions may bind to cysteine 84, inhibiting HINT1 sumoylase activity. Color images are available online.

HINT1 has emerged as a cysteine protease regulated by zinc ions, CaM, and redox processes (Fig. 10), and this regulation is absent in SENPs or DeSIs. HINT1 shows certain homology with matrix metalloproteinases (MMPs) and caspases in which a critical cysteine co-ordinates a zinc ion in the catalytic site that inhibits the enzyme, thus allowing these proteins to be activated by NO (36, 59). Disruption of the zinc-cysteine interaction activates the MMP by a mechanism known as the cysteine switch. The availability of NO to react with the cysteine thiol to form the S-nitrosylated derivative is facilitated by the colocalization of MMP with nNOS (17). Similarly, HINT1 also colocalizes with nNOS in the MOR environment where HINT1 is associated with RGSZ2–nNOS complexes (18). On MOR activation, Gα-GTP subunits bind to the HINT1-bound RGSZ2 protein, provoking nNOS activation and subsequent NO production (48, 50). Thus, as reported for MMP, the proximity of nNOS may facilitate the removal of zinc ions from the HINT1 catalytic Cys84 by NO. The MOR establishes physical interactions with glutamate NMDA calcium ionotropic receptors (42), which activate CaM and nNOS. In this scenario, CaM binding to the HINT1 N-terminal region may remove zinc ions from the catalytic Cys84 and displace desumoylated binding partners, such as RGSZ2 and ICD teneurin1, from their binding to HINT1. Thus, NMDAR activity may provide calcium- and NO-mediated regulation of HINT1 isopeptidase activity.

Initial studies suggested that the linear amino acid sequence containing the HIT was the HINT1 binding zinc site (35). A subsequent study on the HINT1 3D dimeric structure refined the initial idea and proposed His51, His112, and His114 as the best zinc-binding candidates (26), and these histidine residues are mutated in human ARAN-NM. Although HINT1 H112N and H114R mutants were devoid of isopeptidase activity, this function was deregulated in the H51R mutant, suggesting that His51 is essential for the zinc-mediated inhibition of HINT1 activity. In zinc metalloenzymes, zinc ions exhibit picomolar to nanomolar affinity when adopting the tetrahedral geometry co-ordinating the sulfur of cysteine, the nitrogen of histidine, the oxygen of aspartate or glutamate, or a combination (31). However, zinc inhibition of caspase-3 is achieved in the mid-nanomolar to low-micromolar range, suggesting that Zn^2+^ binds to a single or a couple of amino acid residues, probably His237 and Cys285, in the catalytic site. Previous studies reported the binding of a single zinc ion per HINT1 molecule with a Kd of 4.3 μ*M* (35), which suggests that zinc inhibits HINT1 isopeptidase activity by bridging a couple of amino acid residues, probably Cys84 and His51. Thus, the HINT1 catalytic triad may include Cys84 but not Cys38 together with Asp87 and probably His114 as well.

The reported HINT1 purine nucleoside phosphoramidase and adenylate hydrolase activity (8, 11) diminishes in the presence of low-micromolar concentrations of divalent metal ions with a rank order of Cu^2+^ > Zn^2+^ > Cd^2+^ ≥ Ni^2+^ > Mn^2+^, but it is still observed in the presence of chelators such as ethylenediaminetetraacetic acid (53). The isopeptidase activity of HINT1 was also reduced by such a range of divalent metal ion concentrations and with a similar rank order Cu^2+^ > Zn^2+^ > Ni^2+^ > Mn^2+^. Alternatively, mid-nanomolar concentrations of the substrate TpAd and of the inhibitor TpGc of HINT1 adenylate hydrolase activity inhibited HINT1 sumoylase activity. These observations suggest that both enzymatic activities share the substrate recognition site and/or the catalytic site on the HINT1 protein. Although HINT1 nucleotide hydrolysis is strongly diminished in the His114 mutant, this activity remains after a single cysteine substitution, C38A or C84A, with the double cysteine mutant exhibiting 1/10th of the WT activity (38). Therefore, this HINT1 function may require the integrity of His114 but is not as dependent on Cys84 as the isopeptidase activity is. To date, no report has described HINT1 purine nucleoside phosphoramidase and adenylate hydrolase activity to be regulated by calcium, CaM, or redox processes. The affinity of zinc for the HINT1 protein is in the low-micromolar concentration range (35), similar to that required to inhibit its adenylate hydrolase activity (53). It is possible that certain protein purification procedures remove zinc from its inhibitory binding to the HINT1 protein, thus releasing this enzymatic activity.

The HINT1 mutant H112N lacks isopeptidase and adenylate hydrolase activity; however, WT HINT1 and the H112N mutant promoted p53-mediated apoptosis when expressed in SW480 and MCF-7 cells (66). This observation may suggest that some signaling features of the HINT1 protein are independent of its enzymatic activity. Nevertheless, it is possible that these functions must be blocked for HINT1 to promote this signaling. HINT1 establishes zinc-dependent associations with proteins such as PKCγ and Raf-1 and calcium-dependent interactions with σ1R, RGSZ2, and ICD teneurin1 (42, 47, present study), which may, indeed, affect its sumoylase activity. Our *in vitro* assays revealed that the regulatory cytosolic region of the NMDAR NR1 subunit and σ1R abrogated the isopeptidase activity of HINT1, which persisted when HINT1 was bound to MOR, RGSZ2, or ICD teneurin1. This *in vivo* inhibitory regulation of HINT1 desumoylase activity was assessed in brain tissue obtained from mice that received drugs known to disrupt HINT1 interactions with those proteins able to inhibit its isopeptidase function *in vitro*, that is, NMDAR NR1 subunits and σ1Rs (44, 46). The *ex vivo* study confirmed that HINT1 binds to the MOR (42) and that σ1Rs or NMDARs blocked HINT1's access to its substrate, sumoylated RGSZ2. The *in vivo* administration of σ1R antagonists S1RA and BD1063 and the NMDAR agonist NMDA facilitated the association of sumoylated RGSZ2 proteins with MOR–HINT1 complexes (42) and probably the recruitment of HINT1 isopeptidase activity as well.

These observations and previous data delineate how HINT1 isopeptidase activity may be regulated in the neural membrane. In the resting state, the HINT1 protein binds to the MOR C terminal cytosolic sequence (18). In this situation, HINT1 may also bind to NR1 subunits of silent NMDARs, interact with σ1Rs, or form complexes with desumoylated RGSZ2 and inactive PKCγ (1, 44). In the latter scenario, the action of nNOS/NO on RGSZ2 CRD provides zinc ions to couple inactive PKCγ to the HINT1 protein (14, 48). Agonist-induced signaling through MORs promotes the separation of PKCγ from the MOR–HINT1–RGSZ2 complex and activation of the kinase *via* Gβγ-phospholipase C β-calcium/diacylglycerol. The activity of PKCγ in this environment releases RGSZ2–nNOS from the MOR–HINT1 complex, which is now ready to couple with NR1 subunits to regulate the activity of NMDARs. The direct activation of NMDARs or regulators of σ1Rs promotes the separation of active NMDARs from MOR–HINT1 complexes (42, 46). In this scenario, HINT1 binds to sumoylated RGSZ2, and the calcium levels provided by NMDAR function activate CaM and nNOS/NO, which then recruit HINT1 isopeptidase. Thus, Ca^2+^-CaM and NO oppose the inhibitory effect of zinc ions, which are ready to bind to HINT1 Cys84. After the cessation of NMDAR activity, calcium levels diminish and consequently decrease CaM and nNOS activity, and zinc ions can then bind the reduced Cys84 to inhibit HINT1 desumoylase activity.

The interaction of ICD teneurin1 and RGSZ2 with the HINT1 protein may be relevant to the onset of certain diseases. HINT1 facilitates the transportation of these proteins from the plasma membrane to the nucleus to regulate gene transcription. Teneurin1, a protein implicated in Alzheimer's disease, is a transmembrane glycoprotein that is highly expressed in the CNS of mammals, which regulates processes such as the Wnt/β-catenin transcriptional pathway, neurite outgrowth, axon guidance, fasciculation target recognition, and synaptogenesis (6, 69). Because ICD teneurin1 requires HINT1 to promote gene transcriptional regulation, the impaired isopeptidase activity of human HINT1 mutants may alter ICD teneurin1 regulation of gene expression and contribute to triggering ARAN-NM in humans. Interestingly, targeted disruption of the *HINT1* gene does not promote neuropathy-related phenotypes, at least in mice (52). This finding suggests that human HINT1 mutants alter the function of third-partner proteins, such as those included in this study, and thus cause the disease. Indeed, σ1R is enriched in motoneurons (30), and its human mutations have been implicated in distal hereditary motor neuropathies (49), even in devastating amyotrophic lateral sclerosis (ALS) (2). HINT1 collaborates with σ1R to regulate the function of glutamate NMDARs (44), and the progression of ALS is delayed by drugs such as riluzole, which diminishes the function of these NMDARs (28). The HINT1-interacting protein RGSZ2 has also been related to different types of cancer, such as lung, prostate, ovarian, breast, hepatocellular carcinoma, and colorectal cancer (see the section “Introduction”). However, there are no data available on the relevance of the sumoylated RGSZ2 forms in these diseases.

In summary, HINT1 sumoylase activity is inhibited by the binding of zinc ions to Cys84 at the catalytic site and probably to His51 as well. In addition, HINT1's sumoylase activity is inhibited through its interaction with third-partner proteins such as NMDARs and σ1Rs. After disruption of these inhibitory interactions, HINT1 isopeptidase activity is promoted by zinc removal from Cys84 through Ca^2+^-CaM and NO. Thus, redox processes and the interaction of certain signaling proteins regulate this newly discovered activity of the HINT1 protein. The human HINT1 mutants reported to cause ARAN-NM all exhibited deficiencies in their sumoylase activity, thus suggesting a role for HINT1 isopeptidase activity in the pathogenesis of this human motor disease.

## Materials and Methods

### Expression of recombinant proteins

The coding region of murine full-length (1–126) HINT1 (NM_008248.2) and its mutated sequences, HINT2 (NM_026871.1) (1–163), HINT3 (NM_025798.3) (1–165), RGSZ2 (NM_019958.4) (1–210), the ICD region of the Teneurin1 (NM_011855) (residues 2–317), and σ1R (AF004927) (1–223), were amplified by reverse transcription-polymerase chain reaction using total RNA isolated from mouse brains as the template. Specific primers containing an upstream *Sgf*I restriction site and a downstream *Pme*I restriction site were used, as previously described (44). The PCR products were cloned downstream of the GST coding sequence (for RGSZ2 and σ1R) or HaloTag coding sequence (for HINT1, HINT2, HINT3, ICD Teneurin 1), and the TEV protease site. All the sequences were confirmed through automated capillary sequencing, and they were identical to the GenBank™ sequences. The vector was introduced into *Escherichia coli* BL21 (KRX #L3002; Promega), and clones were selected on solid medium containing ampicillin. After 3 h of induction at room temperature (RT) (1 m*M* isopropyl β-D-1-thiogalactopyranoside and 0.1% Rhamnose), the cells were collected by centrifugation, and the pellets were maintained at −80°C.

The GST fusion proteins were purified under native conditions on GStrap FF columns (GE#17-5130-01; GE Healthcare); when necessary, the fusion proteins retained were cleaved on the column with ProTEV protease (#V605A; Promega), and further purification was achieved by high-resolution ion exchange (#780-0001 Enrich Q; BioRad) or electroelution of the corresponding gel band (GE 200; Hoefer Scientific Instruments).

The HaloTag fusion proteins were purified under native conditions with HaloLink Resin (G1915; Promega), and they were cleaved in bulk with ProTEV protease (#V605A; Promega); further purification was achieved by high-resolution ion-exchange chromatography (#780-0001 Enrich Q; BioRad). In a set of assays, HINT1 obtained from a commercial source (Abcam plc; #ab87362) was also used.

To preserve the HINT1, enzymatic activity was essential to keep the HINT1 proteins as dissociable monomers, so their storage plays a critical role as these proteins were found to form dimers, tetramers, and even larger oligomers in solution. The multimeric forms of the HINT1 were not disrupted by disulfide bond reductors or ionic detergents. Thus, for HINT1 WT and its mutated sequences, protein-enriched fractions were concentrated by centrifugation in an Amicon Ultra-0.5 centrifugal filter device (UFC5010BK; Millipore), followed by buffer exchange in PD-10 gel chromatography columns (GE #17-0851-01; GE Healthcare) in a buffer containing 20 m*M* HEPES, pH 7.5, 100 m*M* NaCl, and 1 m*M* dithiothreitol (DTT). HINT1 was concentrated and stored under an inert Argon atmosphere at −80°C.

### Animals, drugs, and metal ions

Male albino CD1 mice, homozygous (σ1R^−/−^) male sigma receptor knockout mice, backcrossed (N10 generation) onto a CD1 albino genetic background (ENVIGO, Milano, Italy), and homozygous (HINT1^−/−^) male HINT1 knockout mice with the genetic background from 129 mice were used in this study. The mice were maintained at 22°C on a diurnal 12 h light/dark cycle. Animals were randomly assigned to each experimental group, and the molecular determinations were performed in naïve mice and those that received the drugs. To facilitate selective and straightforward access to their targets, the compounds were injected (4 μL) into the lateral ventricles of mice. Drug doses and animal per group were selected based on previous work (44, 57). All procedures involving mice adhered strictly to the guidelines of the European Community for the Care and Use of Laboratory Animals (Council Directive 86/609/EEC) and Spanish Law (RD53/2013) regulating animal research. All experiments were approved by the Ethics Committee for Animal Research of CSIC.

The non-competitive inhibitor of HINT1 enzymatic activity TpGc and the HINT1 substrate TpAd were synthetized with iQAC CSIC (Barcelona, Spain). These compounds were initially dissolved in 100% dimethyl sulfoxide (DMSO), and through serial dilutions, the concentrations used in the study were obtained with a final DMSO concentration of ∼0.1%. NMDA (#0114) and BD1063 (#0883) were obtained from Tocris Bioscience (Bristol, United Kingdom); 4-[2-[[5-methyl-1-(2-naphthalenyl)-1H-pyrazol-3-yl]oxy]ethyl] morpholine (S1RA) was obtained from Cayman Chemical United States of America (#16279). NO donors: SNAP (Merk Millipore; #487910), NOR-3 (FK409) (Tocris; #3105), Spermine NONOate (Merk Millipore; #567703), and TPEN (Merk Millipore; #616394). Test drugs were dissolved in saline or DMSO when required.

High-purity divalent metal chloride salts were dissolved in water and evaluated on HINT1 sumoylase activity: CuCl_2_ (Merck-Millipore; #102739), NiCl_2_ (Merck-Millipore; #106717), ZnCl_2_ (Sigma; #39059), MnCl_2_ (Merck-Millipore; #805930), and MgCl_2_ (Sigma; #M8266).

### *In vitro* interactions between recombinant proteins

The recombinant HINT proteins (200 n*M*) or HINT1 mutants were incubated either with Sepharose 4B (GE #17-0120-01; negative control) or together with the immobilized proteins: CaM-agarose 4B (GE Healthcare, GE #17-0529-01), SUMO1-agarose (Boston Biochem; #UL-740), or SUMO2-agarose (Boston Biochem; #UL-755) in 300 μL of a buffer containing 50 m*M* Tris-HCl, pH 7.5, and 0.2% 3-[(3-cholamidopropyl)dimethylammonio]-1-propanesulfonate (CHAPS) in the presence of 2.5 m*M* CaCl_2_ and mixed by rotation for 30 min at RT. After incubation, the pellets were recovered by centrifugation, washed thrice in the presence of 2.5 m*M* CaCl_2_, solubilized in 2 × Laemmli buffer, and analyzed by Western blotting.

The interactions between GST-RanGAP1 (100 n*M*) (Enzo Lifescience; #BML-UW9755), GST-SP100 (100 n*M*) (Enzo Lifescience; #BML-UW9825), and HINT1 (200 n*M*) were studied. In another set of assays, the interactions between GST–HINT1 (100 n*M*) and RGSZ2 (200 n*M*) and ICD teneurin1 (200 n*M*) were analyzed. The proteins were incubated alone (negative control) or together with the GST-tagged protein in 300 μL of a buffer containing 50 m*M* Tris-HCl, pH 7.5, 0.2% CHAPS, and 2.5 m*M* CaCl_2_ and mixed by rotation for 30 min at RT. After incubation, 40 μL glutathione sepharose (GE Healthcare; GE#17-0756-01) was added, and the pellets obtained by centrifugation were washed three times, solubilized in 2 × Laemmli buffer, and analyzed by Western blotting.

### *In vitro* sumoylation and desumoylation assays

*In vitro* sumoylation assays were performed by using the kit provided by Enzo Life Sciences (#BML-UW8955). Briefly, in a 20-μL reaction, 1 μ*M* purified murine recombinant RGSZ2, ICD teneurin1, or GST-RanGAP1 (positive control) was incubated with a reaction mixture containing 50 m*M* Tris-HCl, pH 7.5, 40 m*M* NaCl, 10 m*M* ATP, 10 m*M* MgCl_2_, 100 n*M* SUMO E1, 2 μ*M* SUMO E2, and 50 μ*M* SUMO1 for 1 h at 37°C. To improve the isopeptidase activity of HINT1, after sumoylation of the target protein, the 10 m*M* MgCl_2_ buffer was exchanged with the desumoylation buffer containing 50 m*M* Tris-HCl, pH 7.5, 100 m*M* NaCl, and 0.3 m*M* MgCl_2_ by using a centrifugal filter device (Millipore; 10,000 nominal molecular weight limit, Amicon Ultra-0.5, #UFC5010BK).

Desumoylation assays were carried out by incubating the sumoylated substrate with 2 μ*M* HINT1 in the presence of 6 μ*M* CaM (208670; Calbiochem) plus 2.5 m*M* CaCl_2_, or in the presence of 100 μ*M* NO donor SNAP. SENP2 (300 n*M*, #E-710; Boston Biochem) preincubated with 10 m*M* DTT was used as control of desumoylation. The reactions were performed in a 30-μL reaction for 1 h at 37°C, and they were stopped by adding 2 × Laemmli buffer. The samples were separated by sodium dodecyl sulfate (SDS)/polyacrylamide gel electrophoresis (PAGE) and analyzed by Western blotting.

### Immunoprecipitation and Western blotting

Mice cerebral cortices were obtained and processed to obtain the synaptosomal pellet, and they were used for MOR immunoprecipitation. This procedure has been described elsewhere (13). Briefly, for immunoprecipitation studies, the cortices from eight mice were typically pooled; the assays were repeated at least twice on samples that had received an identical treatment and were collected at the same interval post-administration. The affinity-purified immunoglobulin Gs (IgGs) against the extracellular domains of the MOR second external loop (205–216: MATTKYRQGSID; GenScript Co.) were labeled with biotin (Pierce; #21217 and 21339). The immunocomplexes were recovered and resolved with SDS-PAGE electrophoresis in 10 cm × 10 cm × 1.5 mm gel slabs (7%–14% total acrylamide concentration, 2.6% bisacrylamide cross-linker concentration). Separated proteins were transferred onto 0.2 μm polyvinylidene difluoride membranes (Bio-Rad; #162-0176) and probed overnight at 6°C with the selected primary antibodies diluted in Tris-buffered saline (TBS; pH 7.7) + 0.05% Tween 20 (TTBS). These proteins were detected by using secondary antibodies conjugated to horseradish peroxidase. The secondary antibodies were directed to either the heavy or light IgG chains of the primary antibodies as needed to preserve the target immunosignal. In parallel gel blots loaded with a fraction of the samples, the immunoprecipitated MORs were detected. Because the secondary antibodies reacted primarily with the IgG light chains of the primary and the accompanying antibodies used for immunoprecipitation of MOR, these signals when needed also provided a loading control for the samples in the gel.

The *in vitro* assays using recombinant proteins did not require immunoprecipitation; thus, IgGs were excluded. In protein interaction studies, cloned proteins such as SUMO and CaM were immobilized through covalent attachment to N-hydroxysuccinimide-activated Sepharose 4 FF, carried GST that was pulled down by using agarose-glutathione. Subsequently, the target recombinant proteins, for example HINT1 and mutants, were coincubated with agarose-immobilized proteins or GST proteins. Then, the agarose containing the protein complexes went through repeated centrifugation-washing cycles. Next, target proteins were detached from agarose-immobilized proteins by using SDS Laemmli buffer, resolved by SDS-PAGE and detected by Western blotting with the appropriate antibodies. Thus, the blot areas containing the corresponding sizes of the cloned target proteins were selected for image capture and analysis. In sumoylation assays, the sumoylated recombinant protein was identified through anti SUMO antibodies and specific anti-target proteins when available, for example RGSZ2 and ICD teneurin1. The effect of HINT1 and SENP on RanGAP1 and RGSZ2 sumoylated proteins is shown. For ICD teneurin1, the bands initially sumoylated are shown.

The Western blot images and antibody binding were visualized by chemiluminescence (Bio-Rad; #170-5061) and recorded by using an ImageQuant™ LAS 500 (GE). For each blot, the area containing the target protein was typically selected (with the exception of *ex vivo* assays, which included a wider area of protein sizes). The device automatically captures the selected area and calculates the optimal exposure time to provide the highest possible signal to enable accurate quantification of the sample. Protein immunosignals were measured by using the area of the strongest signal of each studied group of samples (average optical density of the pixels within the object area/mm^2^; AlphaEase FC software). The gray values of the means were then normalized within the 8 bit/256 gray levels [(256 − computed value)/computed value].

### Antibodies

The primary antibody to detect immunoprecipitated receptor was: anti-MOR Ct aa 387–398 (GenScript Co.). Other primary antibodies used in this study were: anti-CaM (Millipore; #05-173), anti-GST (Cell Signaling; #2622), anti-HINT2 (Abnova; #H00084681-01), anti-RGSZ2(1) (Thermo Scientific; #PA1-25695), anti-RGSZ2(2) (aa 192-215; GenScript Co.) (14), anti-teneurin1 (Novus Biologicals; #NBP2-41315), anti-σ1R (Invitrogen; #42-3300), anti-SUMO1 (Enzo; #BML-PW9460), and anti-SUMO-2/3 (Enzo; #BML-PW9465). The anti-HINT1 antibody was raised in rabbits (Immunostep) against the peptide sequence GYRMVVNEGADGGG (93–106). All primary antibodies were detected by using the appropriate horseradish peroxidase-conjugated secondary antibodies.

### Statistical analysis

All graphs and statistical analyses were generated and performed by using the Sigmaplot/SigmaStat v.14 package (SPSS Science Software; Erkrath). Experiments were performed in triplicate on separate experimental days. Data using recombinant proteins and from *ex vivo* protein determinations were analyzed by using one-way analysis of variance (ANOVA) followed by the Holm-Sidak multiple-comparisons test. Data from divalent cation effects were analyzed by using two-way ANOVA, with ion and concentration as main factors. All experiments produced a significant interaction; thus, the follow-up analysis involved one-way ANOVA followed by Holm-Sidak multiple-comparisons test. The effect of TpGc and of TpAd on HINT1 isopeptidase activity was analyzed by nonlinear regression-ligand binding-one site competition. Statistical significance was defined as *p* < 0.05.

## Supplementary Material

Supplemental data

Supplemental data

Supplemental data

Supplemental data

Supplemental data

Supplemental data

Supplemental data

Supplemental data

Supplemental data

Supplemental data

## Abbreviations Used

3D: three-dimensional
ALS: amyotrophic lateral sclerosis
ANOVA: analysis of variance
ARAN-NM: autosomal recessive axonal neuropathy with neuromyotonia
BME: β-mercaptoethanol
CaM: calmodulin
CNS: central nervous system
CRD: cysteine-rich domain
DeSI: desumoylating isopeptidase
DMSO: dimethyl sulfoxide
GPCR: G protein-coupled receptor
GST: glutathione *S*-transferase
HINT1: histidine triad nucleotide-binding protein 1
HIT: histidine triad
ICD: intracellular domain
icv: intracerebroventricular
IgG: immunoglobulin G
MMP: matrix metalloproteinase
MOR: mu-opioid receptor
NMDA: *N*-methyl-d-aspartate
NMDAR: *N*-methyl-d-aspartate receptor
nNOS: neural nitric oxide synthase
NO: nitric oxide
NOR-3: (2*E*,3*E*)-4-ethyl-2-(hydroxyimino)-5-nitro-3-hexeneamide
PAGE: polyacrylamide gel electrophoresis
PKC: protein kinase C
RanGAP1: Ran GTPase-activating protein 1
RGSZ2: regulator of G protein signaling 17 (Z2)
RT: room temperature
SDS: sodium dodecyl sulfate
SE: standard error
SENP: sentrin-specific protease
SIM: SUMO-interacting motif
SNAP: *S*-Nitroso-*N*-acetyl-dl-penicillamine
SUMO: small ubiquitin-like modifier
TBS: Tris-buffered saline
TpAd: phosphoramidate tryptamine AMP
TPEN: *N*,*N*,*N*′,*N*′-tetrakis(2-pyridylmethyl) ethylenediamine
TpGc: guanosine-5′-tryptamine carbamate
WT: wild type
