# Supplementary material for: The Axonal Motor Neuropathy-Related HINT1 Protein Is a Zinc- and Calmodulin-Regulated Cysteine SUMO Protease
Source: Antioxid Redox Signal. 2019 Jul 17;31(7):503–20. doi: 10.1089/ars.2019.7724 (PMC6648240; doi:10.1089/ars.2019.7724)
Supplement: Supplemental data [file Supp_Figure4.pdf]

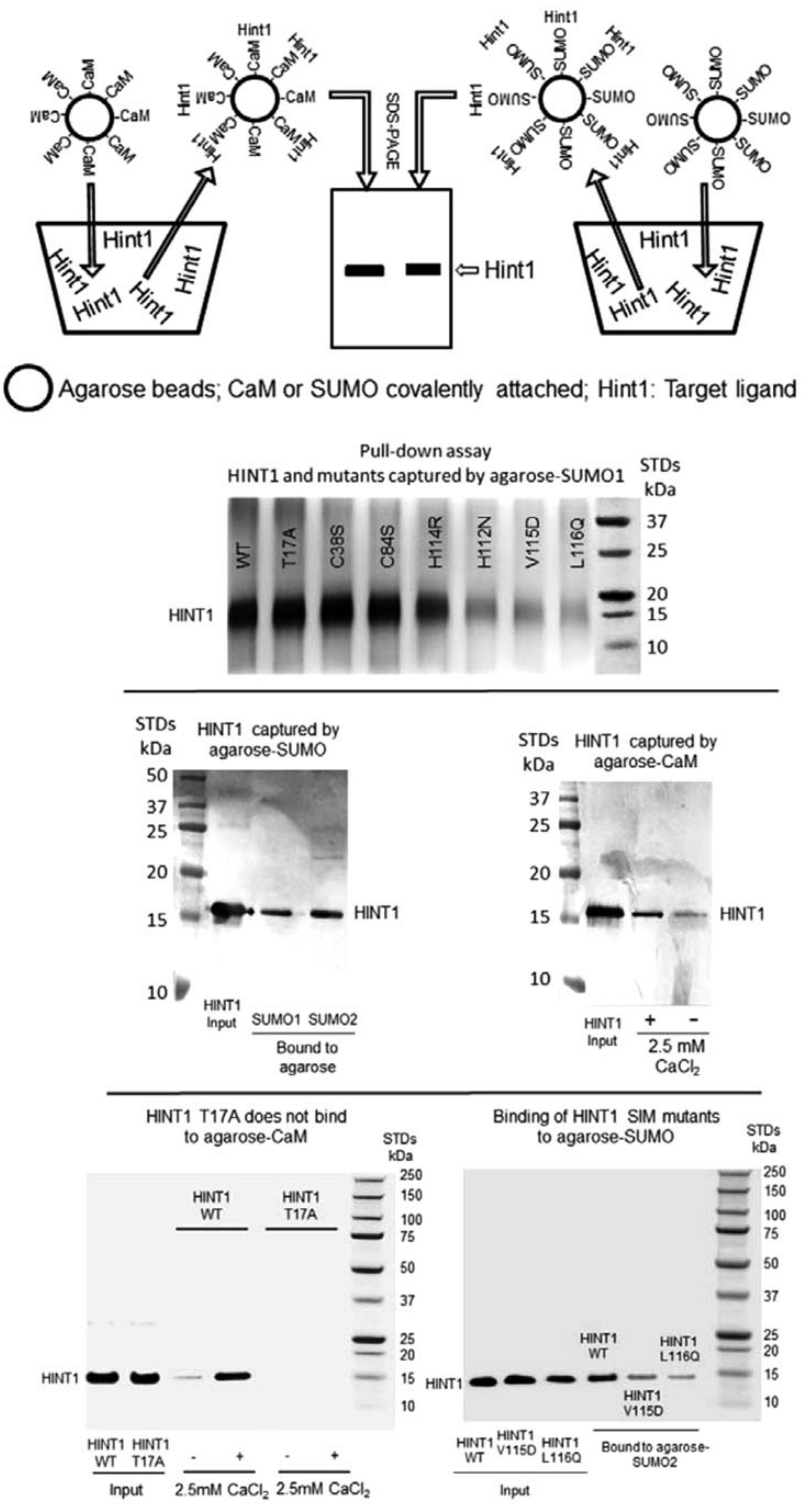


**SUPPLEMENTARY FIG. S4. Typical pull-down assays.** The Western blot images, antibody binding, were visualized by chemiluminescence and recorded by using an ImageQuant™ LAS 500 (GE). In the original blot the STDs were colored but the studied proteins were revealed through chemiluminescence, and the capture device combined protein data and STDs images. Because the assays performed with recombinant proteins produce the expected protein sizes, the capture of the data was optimized. Thus, for each blot, the area containing the recombinant protein was typically selected. The device automatically captures the selected area and calculates the optimal exposure time to provide the highest possible signal to enable accurate comparison between the samples. For quantification, for example, Figures 5 and 7, protein immunosignals were measured by using the area of the strongest signal of each studied group of samples (AlphaEase FC software). STD, protein size standards.
