# Supplementary material for: The Axonal Motor Neuropathy-Related HINT1 Protein Is a Zinc- and Calmodulin-Regulated Cysteine SUMO Protease
Source: Antioxid Redox Signal. 2019 Jul 17;31(7):503–20. doi: 10.1089/ars.2019.7724 (PMC6648240; doi:10.1089/ars.2019.7724)
Supplement: Supplemental data [file Supp_Figure1.pdf]

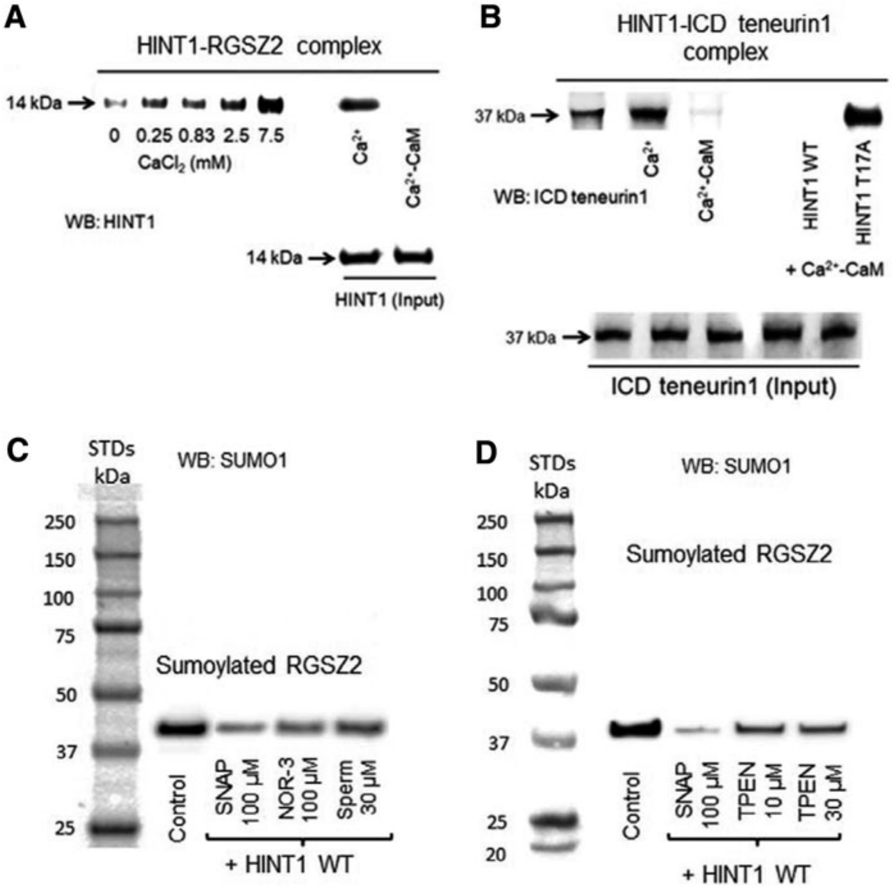

SUPPLEMENTARY FIG. S1. Calcium promotes HINT1 stable complexes with RGSZ2 and ICD teneurin1, and zinc inhibits HINT1 isopeptidase activity. **(A)** CaM and RGS17(Z2) share the HINT1 interacting surface. HINT1–RGSZ2 association increases in the presence of $CaCl_2$. In physiological 2.5 m*M* calcium, CaM competed with RGSZ2 for HINT1 binding. Wild-type HINT1 (100 n*M*), RGSZ2 (200 n*M*), $CaCl_2$ (2.5 m*M*), and CaM (6 *μM*). **(B)** CaM and ICD teneurin1 share the HINT1 interacting surface. The association of HINT1 with ICD teneurin1 increased in the presence of physiological calcium. CaM and ICD teneurin1 competed to bind to HINT1. The T17A HINT1 mutant was unable to bind CaM but still bound ICD teneurin1. Wild-type HINT1 and T17A (100 n*M*), ICD teneurin1 (200 n*M*), $CaCl_2$ (2.5 m*M*), and CaM (6 *μM*). **(C, D)** The NO donors SNAP, NOR-3, and spermine NONOate, and the heavy metal chelator TPEN used at the concentrations indicated promoted HINT1 isopeptidase activity on sumoylated RGSZ2. The assays were performed at least twice, and each point was duplicated. Wild-type HINT1 (2 *μM*), RGSZ2 (1 *μM*). CaM, calmodulin; HINT1, histidine triad nucleotide-binding protein 1; ICD, intracellular domain; NOR-3, (2*E*,3*E*)-4-ethyl-2-(hydroxyimino)-5-nitro-3-hexeneamide; RGSZ2, regulator of G protein signaling 17 (Z2); SNAP, *S*-nitroso-*N*-acetyl-DL-penicillamine; SUMO, small ubiquitin-like modifier; TPEN, *N*,*N*,*N′*,*N′*-tetrakis(2-pyridylmethyl) ethylenediamine.
