# Supplementary figures and images for: The Axonal Motor Neuropathy-Related HINT1 Protein Is a Zinc- and Calmodulin-Regulated Cysteine SUMO Protease
Source: Antioxid Redox Signal. 2019 Jul 17;31(7):503–20. doi: 10.1089/ars.2019.7724 (PMC6648240; doi:10.1089/ars.2019.7724)

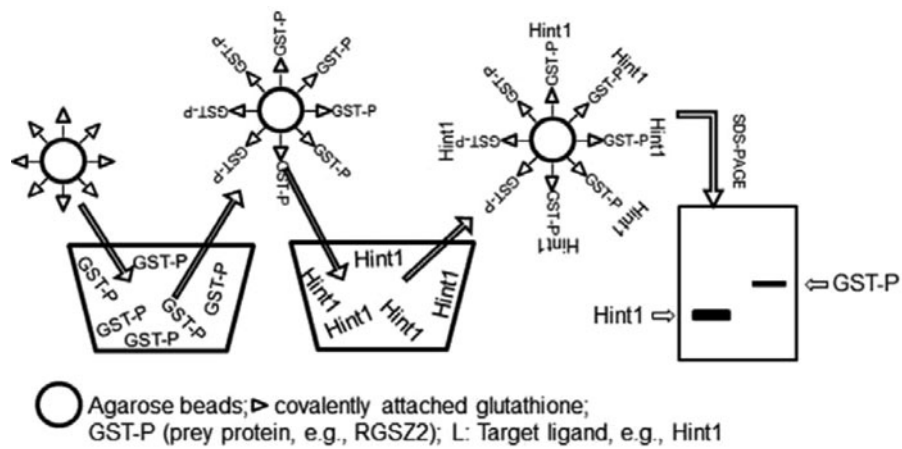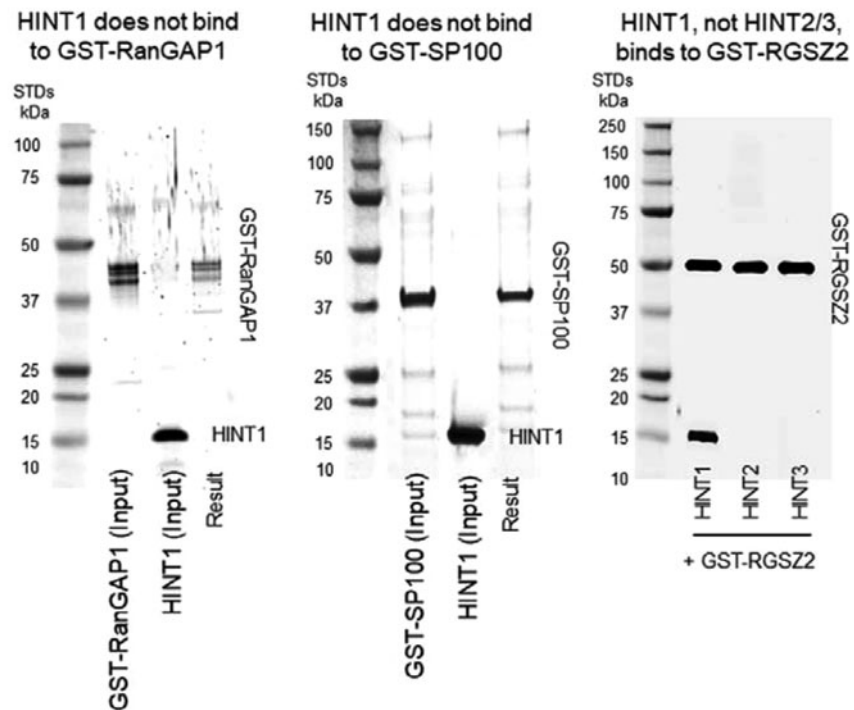

**SUPPLEMENTARY FIG. S5. Pull-down assays, GST variant.** GST, glutathione *S*-transferase.

Supplement: Supplemental data [file Supp_Figure5.pdf]
