# Supplementary material for: The Axonal Motor Neuropathy-Related HINT1 Protein Is a Zinc- and Calmodulin-Regulated Cysteine SUMO Protease
Source: Antioxid Redox Signal. 2019 Jul 17;31(7):503–20. doi: 10.1089/ars.2019.7724 (PMC6648240; doi:10.1089/ars.2019.7724)
Supplement: Supplemental data [file Supp_Figure6.pdf]

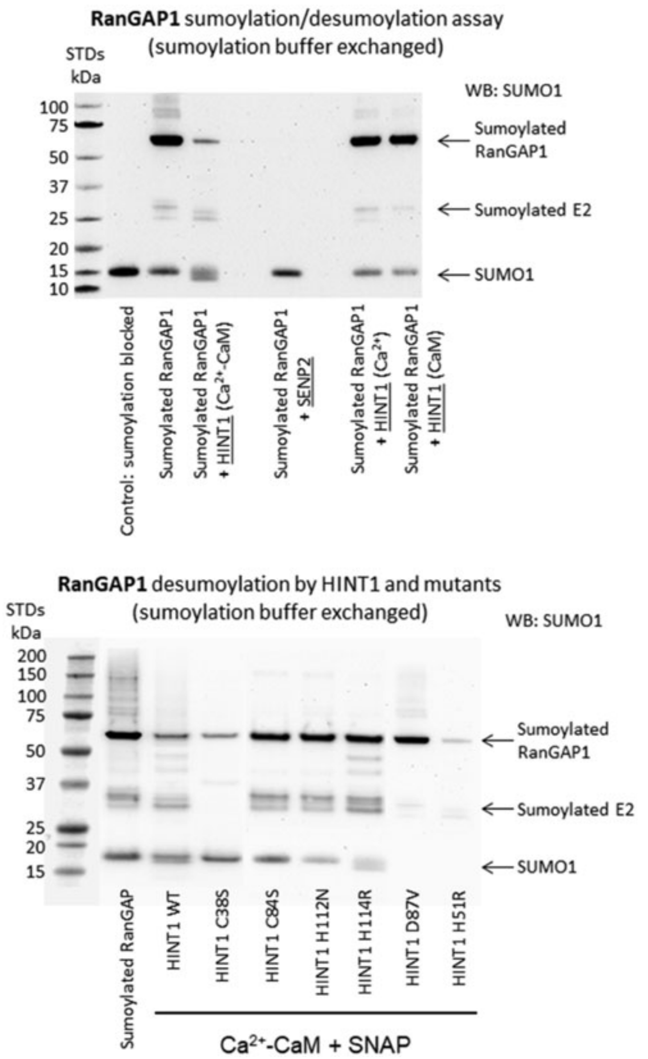

SUPPLEMENTARY FIG. S6. Typical RanGAP1 assays. Desumoylation of recombinant RanGAP1 by HINT1 and SENP. In the main figures, the bands corresponding to the sumoylated RanGAP1 are shown. Details in ''Materials and Methods'' section and ''Immunoprecipitation and Western blotting'' and Supplementary Figure S4. RanGAP1, Ran GTPase-activating protein 1.
