# Supplementary material for: The Axonal Motor Neuropathy-Related HINT1 Protein Is a Zinc- and Calmodulin-Regulated Cysteine SUMO Protease
Source: Antioxid Redox Signal. 2019 Jul 17;31(7):503–20. doi: 10.1089/ars.2019.7724 (PMC6648240; doi:10.1089/ars.2019.7724)
Supplement: Supplemental data [file Supp_Figures2-3.pdf]

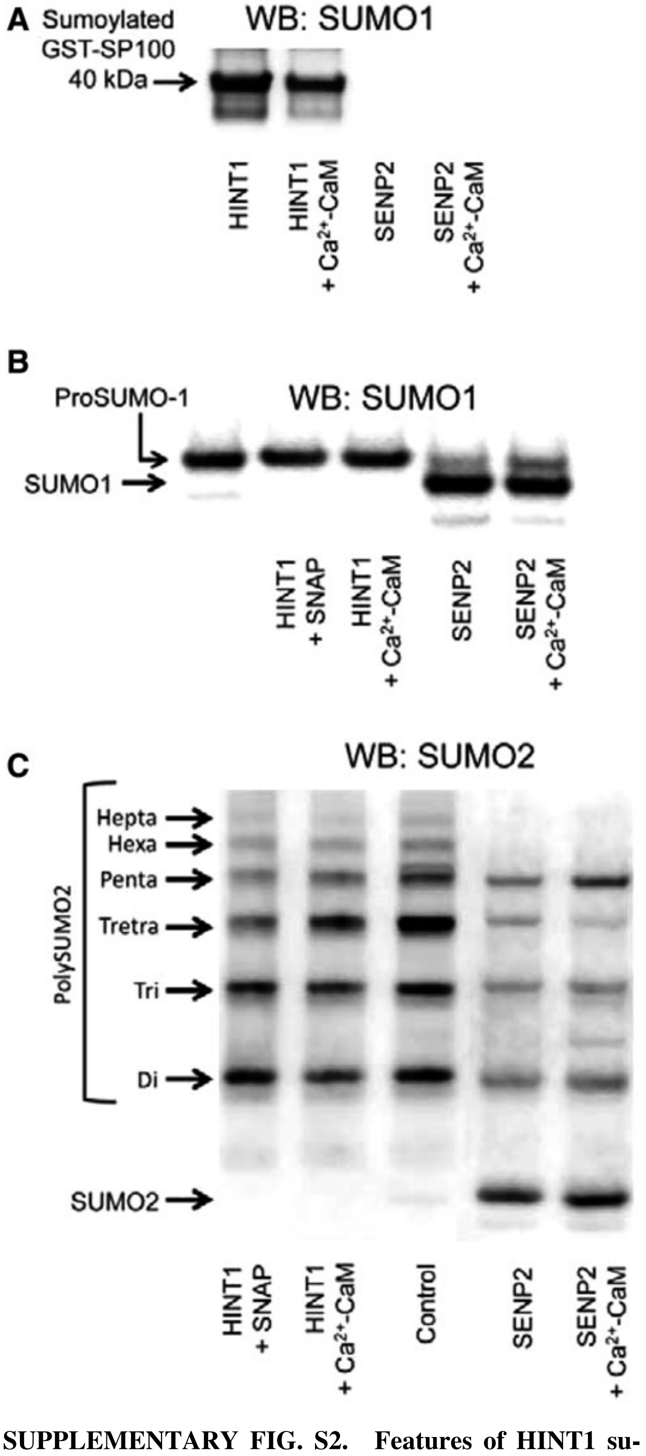

SUPPLEMENTARY FIG. S2. Features of HINT1 sumoylase capacity. (A) SENP2 but not HINT1 cleaved SUMO from SP100 protein, **(B)** from maturated proSUMO, **(C)** and cleaved polySUMO2 chains. Assays were performed in the absence and presence of Ca^{2+}-CaM. HINT1 (2 μM), SENP2 (0.3 μM), SNAP (100 μM), $CaCl_2$ (2.5 mM), and CaM (6 μM). SENP, sentrin-specific protease.

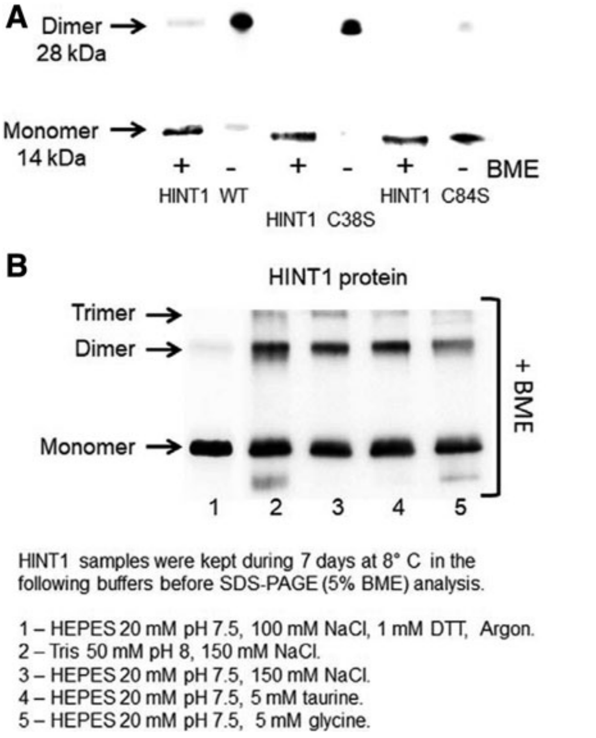

SUPPLEMENTARY FIG. S3. Storage of cloned HINT1 to preserve its sumoylase activity. (A) HINT1 forms Cys84-dependent disulfide bridges between protomers. **(B)** Stability of the HINT1 monomer in different buffers. BME, *β*-mercaptoethanol; SDS-PAGE, sodium dodecyl sulfate-polyacrylamide gel electrophoresis.
