# Supplementary material for: The Axonal Motor Neuropathy-Related HINT1 Protein Is a Zinc- and Calmodulin-Regulated Cysteine SUMO Protease
Source: Antioxid Redox Signal. 2019 Jul 17;31(7):503–20. doi: 10.1089/ars.2019.7724 (PMC6648240; doi:10.1089/ars.2019.7724)
Supplement: Supplemental data [file Supp_Figure7.pdf]

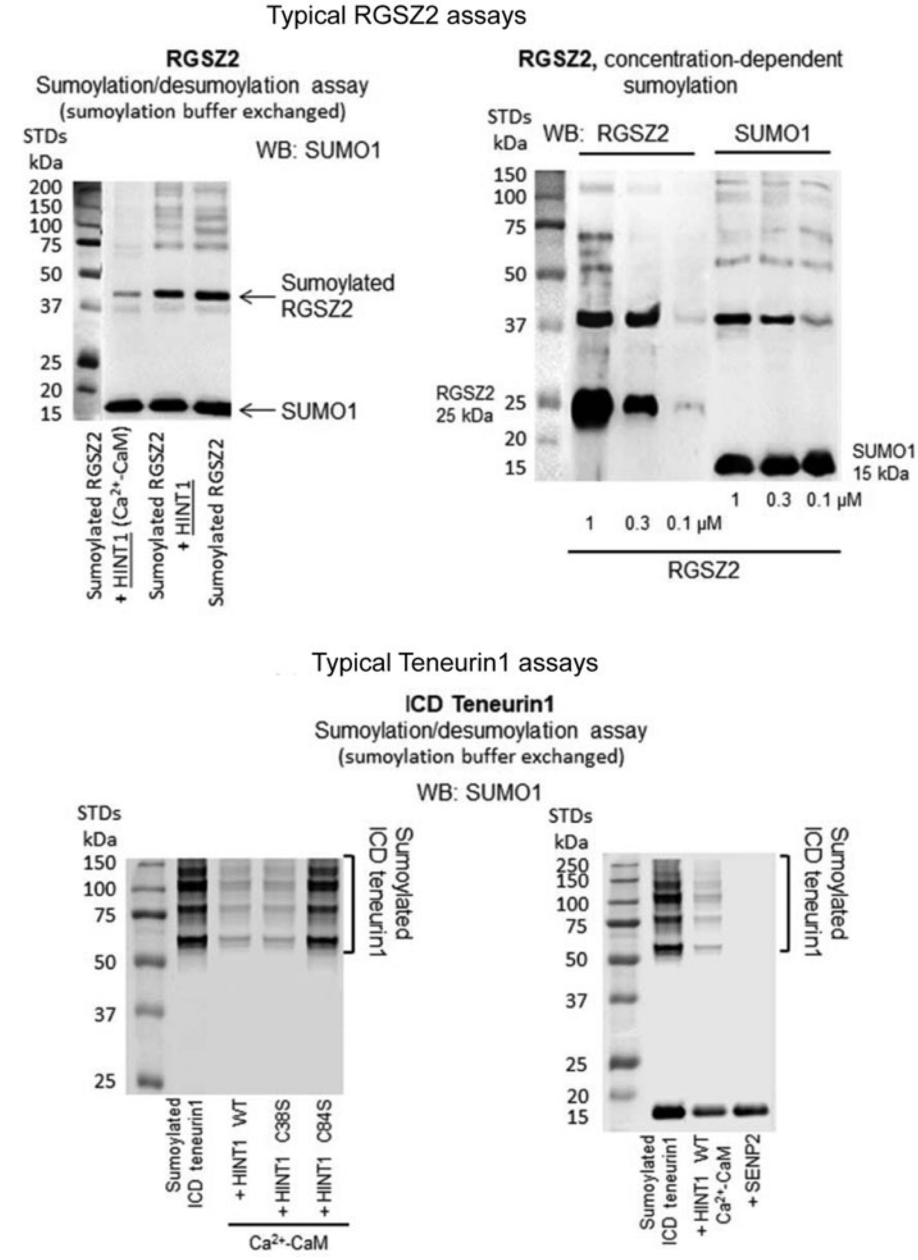

SUPPLEMENTARY FIG. S7. Typical RGSZ2 and ICD teneurin1 assays. Sumoylation concentration-dependent of RGSZ2. Desumoylation of these recombinant proteins by HINT1. In the main figures, the 37 kDa band of sumoylated RGSZ2 is typically shown; however, teneurin1 sumoylated sizes are shown. Details in ‘‘Materials and Methods’’ section and ‘‘Immunoprecipitation and Western blotting’’ and Supplementary Figure S4.
