# Supplementary material for: The Axonal Motor Neuropathy-Related HINT1 Protein Is a Zinc- and Calmodulin-Regulated Cysteine SUMO Protease
Source: Antioxid Redox Signal. 2019 Jul 17;31(7):503–20. doi: 10.1089/ars.2019.7724 (PMC6648240; doi:10.1089/ars.2019.7724)
Supplement: Supplemental data [file Supp_Figure9.pdf]

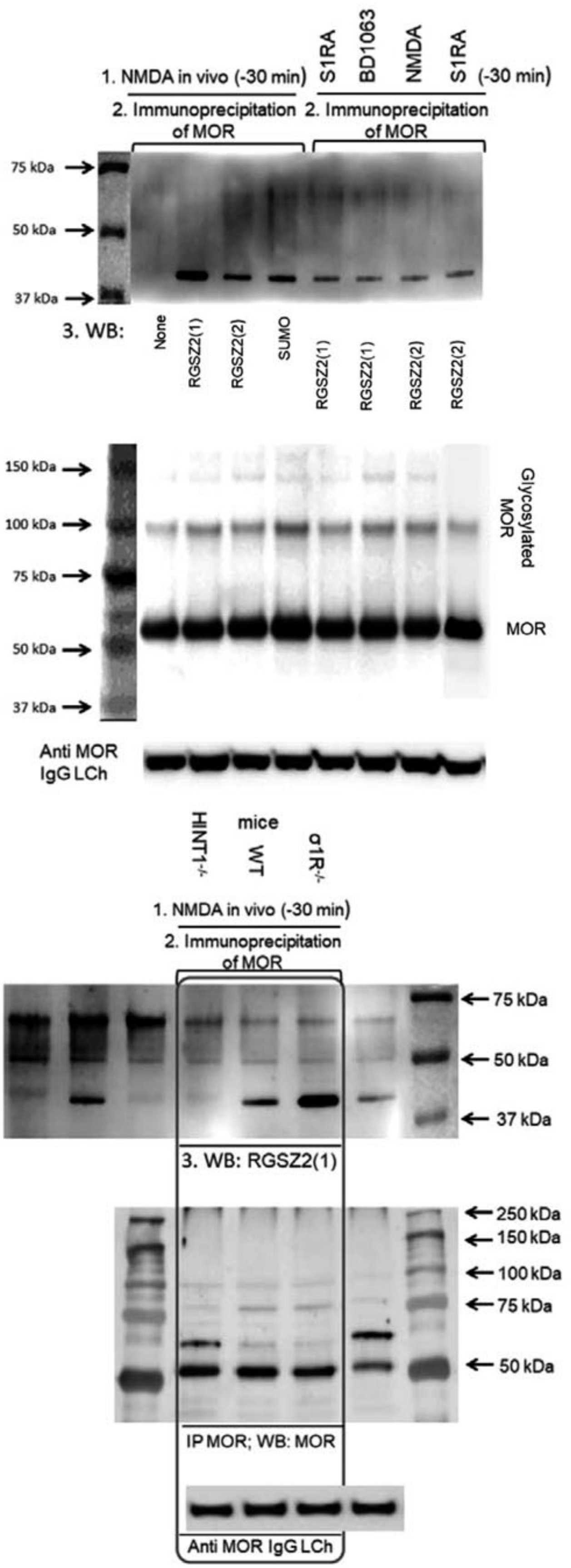

SUPPLEMENTARY FIG. S9. ***Ex vivo*** **detection of MOR-associated sumoylated RGSZ2 protein.** The mice received the drugs *in vivo*, and after sacrifice, the MOR was immunoprecipitated, and associated RGSZ2 protein was determined. Details in the main text and ''Materials and Methods'' section. The studied range of protein sizes permitted accurate detection of the target proteins. Determination of the immunoprecipitated MOR and LCh of the IgGs used served as loading controls. In the *lower panel*, the *rectangle* indicates the data of the assay presented in Figure 8 in the main text. IgG, immunoglobulin G; LCh, light chain; MOR, mu-opioid receptor.
