# Supplementary material for: The Axonal Motor Neuropathy-Related HINT1 Protein Is a Zinc- and Calmodulin-Regulated Cysteine SUMO Protease
Source: Antioxid Redox Signal. 2019 Jul 17;31(7):503–20. doi: 10.1089/ars.2019.7724 (PMC6648240; doi:10.1089/ars.2019.7724)
Supplement: Supplemental data [file Supp_Figure10.pdf]

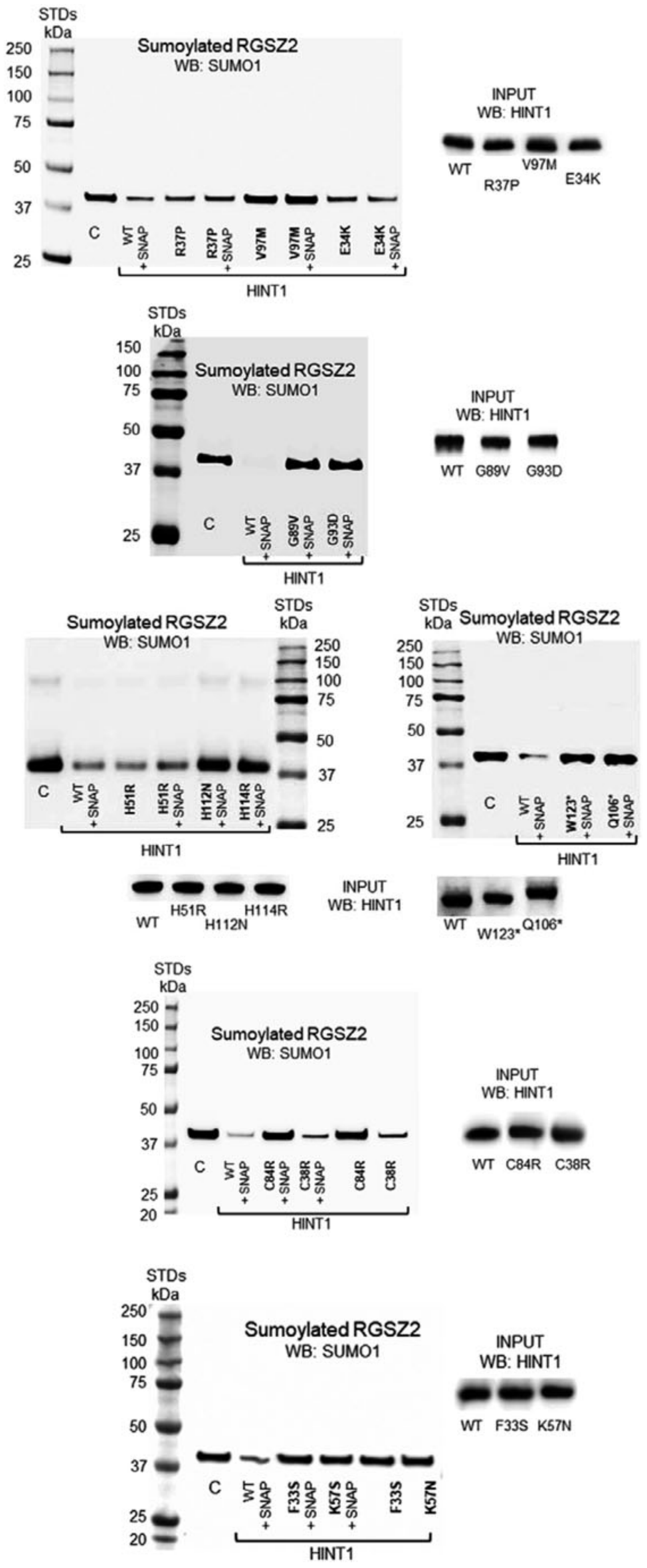

SUPPLEMENTARY FIG. S10. Isopeptidase activity of human HINT1 mutants that cause ARAN-NM. For details, see Figure 9 in main text. ARAN-NM, autosomal recessive axonal neuropathy with neuromyotonia.
