# Supplementary material for: The Axonal Motor Neuropathy-Related HINT1 Protein Is a Zinc- and Calmodulin-Regulated Cysteine SUMO Protease
Source: Antioxid Redox Signal. 2019 Jul 17;31(7):503–20. doi: 10.1089/ars.2019.7724 (PMC6648240; doi:10.1089/ars.2019.7724)
Supplement: Supplemental data [file Supp_Table1.pdf]

Supplementary Data

Supplementary Table S1. HINT1 Mutants Not Related to Human Autosomal Recessive Axonal Neuropathy with Neuromyotonia Included in the Study

HINT1	*Regulation*		*Isopeptidase activity*	*Target*
	Ca2+-CaM	*NO*		
Wild type	Y	Y	Y	
T17A	N	Y	Y	CaM-motif
C38S	Y	Y	Y	
C84S	N	N	N	Catalytic site
D87V	N	N	N	Catalytic site
V115D	N	N	N	SIM
L116Q	ND	ND	ND	SIM

The columns describing isopeptidase activity and its regulation: N and Y indicate No and Yes, respectively.

CaM, calmodulin; HINT1, histidine triad nucleotide-binding protein 1; ND, nondetermined; NO, nitric oxide; SIM, SUMO-interacting motif.
